# Versatility of TiO_2_ Nanoparticles Surfaces in ACN, DMSO, and Aqueous Natural Secretions: Colloidal Behavior

**DOI:** 10.1021/acsomega.6c02670

**Published:** 2026-05-18

**Authors:** Anna Laguta

**Affiliations:** † 52735 University of Chemistry and Technology Prague, Technická 5, 166 28 Prague 6, Czech Republic; ‡ V. N. Karazin Kharkiv National University, Svoboda Square 4, 61022 Kharkiv, Ukraine

## Abstract

The versatile physical properties of core–shell TiO_2_ nanoparticles, compared to bulk titania polymorphs, create a synthesis challenge for highly dispersed particles. This strategy leads to true colloids whose behavior in fluids differs from that of the two-phase systems. The large specific surface area produces a high excess surface energy of the system and the effects it generates. The behavior of nanotitania in aqueous systems must be considered in the application strategy, as the production scale is significant and raises concerns about toxicity and pollution. Researchers are currently focusing on the methods of production, surface modifications, and the impact of morphology on toxicity in biological systems. Colloidal stability defines the applications of nanoparticles in aqueous and nonaqueous systems, including biological fluids as a special case. pH-dependent surface charge accounts for colloidal instability in distilled water (pH = 5.8). These conditions served as the starting point for this work. Medical applications are what prompted the implementation of DMSO as a dispersant. Acetonitrile completed the protocol. The steric hindrance technique involving biological secretion components was applied in an aqueous system. Successful dispersion was achieved after the addition of lysozyme, and it was quite good in the presence of bile salts. The resulting dispersions were subjected to coagulation with electrolytes to examine the effects of in vivo and in vitro cocompounds. Empirical data provide a rationale for strategies of surface coating to facilitate colloidal stability, biocompatibility, and ecological concern for further conjugation with other molecules, such as targeting molecules, drugs, polymers, etc.

## Graphical abstract

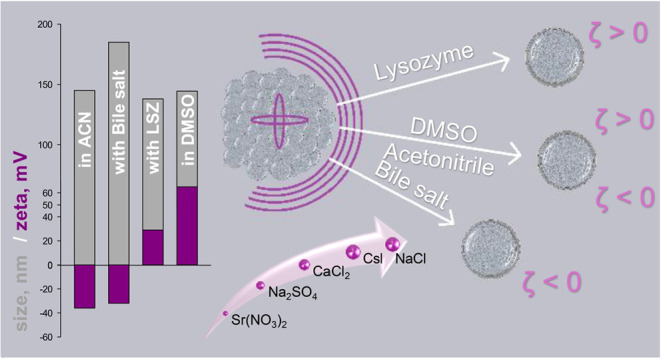

## 1 Introduction

Generally, TiO_2_ has shown promising potential in biological engineering, such as antimicrobial agents, drug delivery systems, photodynamic therapy, biosensors, and biomaterials; it is widely used as food and cosmetic supplements as well as in wastewater treatment.^1–3^

Especially, titania is recognized as a potential photocatalyst.^4^ The light absorption by electrons in the valence band of TiO_2_ generates the electron–hole pairs: the excited electron jumps to the conduction band, forming the hole in the valence band. For example, two of these electrons can promote the reduction of oxygen, and the holes can oxidize the adsorbed water.^5^ Bulk TiO_2_ polymorphs have band gaps covering a small range of relatively high energy solar photons (≥3 eV)^6^ giving rise to low photoefficiencies (only ∼10% of the photons from the sunlight reaching the Earth’s surface).^7^

The generation of intrinsic defects, such as oxygen vacancies, while maintaining the intrinsic activity of the material, led to core–shell nanomorphologies with new physical properties due to the confined geometry of the propagation of quantum excitations.^7^ This anatase-engineered “black TiO_2_” nanospecies has reduced band gaps due to shell-induced band edge broadening, which also displays enhanced photoactivity.^7^

A variety of components, including metal cations (Ca^2+^, Mg^2+^, Fe^3+^, Zn^2+^, and Cu^2+^) and anions or dissolved organic matter, have been found to substantially impact the photocatalytic degradation of the organic pollutants. Their adsorption onto TiO_2_ surfaces alters the surface properties and affects photocatalytic efficiency.^8^

Employing colloidal TiO_2_ in sunscreens, soaps, toothpaste, and paints stimulates large-scale production. The annual global production of TiO_2_ for scientific and industrial purposes is predicted to reach 2.5 million tons,^9,10^ for example, compared to several thousand tons for carbon nanotubes.^11^ The utilized TiO_2_ inevitably reaches aquatic systems via wastewater effluent. For example, toothpaste is projected to contribute 4100 tons of TiO_2_ to wastewater annually in the United States.^12^ The TiO_2_ effects upon the marine dominant cyanobacterium and natural marine communities were examined in ref 10; the extensive aggregation behavior of TiO_2_ in saline media involving entrapment of microbial cells induces cell declines of Prochlorococcus cultures. In ref 9, nano-TiO_2_ was reported to disrupt the cell cycle in vitro. TiO_2_ (<30 nm, anatase) was found to have better cytotoxicity against cancer cells and bacteria than other forms.^13^ The results in ref 14 indicate that the erythrocytes abnormally sedimentate under nano-TiO_2_ and are totally different from those treated by micro-TiO_2_. The influence of TiO_2_ nanospecies on the thermal stability and conformation of native DNA under UV irradiation was analyzed in refs 5,15 . Overall, the interaction of nanoparticles with in vivo and in vitro co-components is an important issue because the effects on the colloidal behavior of nanoparticles arise.

Lysozyme (LSZ) with intrinsic antibacterial activity was applied for coatings on titanium foil appropriate for dental implants.^16^ The morphology and surface hydroxyl groups of TiO_2_ nanofibers affect LSZ adsorption and conformational changes.^17^ Polyacrylic acid-modified TiO_2_ displayed enhanced LSZ adsorption compared to pristine TiO_2_.^18^ Taurocholic acid adsorbs onto TiO_2_ but strongly desorbs when it is not present in the bulk phase, and glycocholic acid irreversibly binds.^19^

Thus, established industries for the consumption of nanotitania have emerged. High consumption is linked to the recycling and stability of systems; both areas present specific challenges for nanosystems and remain largely unresolved. A particular issue is contact with biological systems, as well as with the application and disposal. The behavior of nontoxic materials when switching to the nanostate may give rise to implicit negative consequences. The study aims to investigate the colloidal stability of these nanoparticles in organic solvents and aqueous systems containing components of biosecretions.

The paper is organized as follows: (i) discussion of the stability of hydrophobic nanoparticles in aqueous systems; (ii) consideration of acid–base effects of the dispersant using dynamic and electrophoretic light scattering; (iii) analysis of the colloidal stability of LSZ-coated nanospecies and their interactions with NaCl, CaCl_2_, Na_2_SO_4_, and sodium cholate, NaCh; (iv) analysis of the colloidal stability of NaCh-coated nanoparticles and their interactions with NaCl, CsI, CaCl_2_, Sr­(NO_3_)_2_, and LSZ; and (v) analysis of the colloidal stability of nanospecies coated with sodium deoxycholate (NaDCh) and their interactions with NaCl and CaCl_2_. Both solvents and biological secretions can impart a formal charge on the surface, creating an electrical double layer. Referring to the research approach, analysis of colloidal stability is based on the particle hydrodynamic size and ζ-potential as a good index of the interaction magnitude between colloidal particles; an interaction between nanospecies and an electrolyte is based on coagulation kinetics data. The concept is that interactions with biological secretions and electrolytes are described by two mechanisms affecting the stability of hydrophobic colloids: (i) steric repulsion, preventing contact of particle surfaces at close distances, and (ii) electrolyte coagulation by compression of the thickness of the electric double layer.^20,21^ These approaches and techniques were previously applied to the investigation of nanotubes,^22,23^ nanodiamonds,^24,25^ and La–Sr perovskite Manganite nanoparticles.^26^ State-of-the-art experimental techniques, namely, dynamic and electrophoretic light scattering, are involved as powerful methods for coagulation kinetics investigation. The hydrodynamic size of particles is estimated by measuring the fluctuation in scattering intensity and relating this fluctuation to the Brownian motion using a Zetasizer Nano series instrument with dispersion technology and light scattering software.^27–29^ Electrophoretic mobility is estimated by measuring phase change in the scattered light due to the directional movement of colloidal particles in an applied electric field.^30^

## 2 Experimental Section

### 2.1 Materials

The TiO_2_ nanopowder with particle size (BET) of <100 nm and trace metals basis of 99.5% according to the information from the vendor (Sigma-Aldrich, Product code: 677469); lysozyme from chicken egg white powder (crystalline) with enzymatic activity of ≥70000 U/mg (Sigma-Aldrich); sodium deoxycholate (NaDCh, 97% of the main substance, Sigma-Aldrich); sodium cholate (NaCh, 97% of the main substance, Sigma-Aldrich); NaCl, CaCl_2_·2H_2_O, and Na_2_SO_4_·10H_2_O were used as received.

DMSO and acetonitrile were purified via conventional procedures and contained 0.5 and 0.001 mol % water, respectively, as estimated by Reichardt’s standard dye-based solvent polarity (λ_max_ = 633 and 618 nm, respectively, Figure S1).

### 2.2 The Particle Size, ζ-Potential, Surface Charge Density, and Critical Coagulation Concentration Determination Procedure

The hydrodynamic size and electrophoretic mobility of particles in nanofluids were determined by dynamic and electrophoretic light scattering (DLS and ELS, Zetasizer Nano ZS, Malvern Instrument apparatus) at 25 °C. The electrokinetic potential was calculated by the Henry equation (eq 1) using the Hückel–Onsager model (*f* = 1) in salt-free systems and the Ohshima approximation (eq 2) in salt systems.^31^

1 $$\zeta =u_{e}\times \frac{3\times \eta }{2\times \varepsilon_{r}\times \varepsilon_{0}}\times \frac{1}{f}$$

2 $$f=1+0.5\times {\left[1+\frac{2.5}{\kappa \times r\times \left(1+2\times e^{-\kappa \cdot r}\right)}\right]}^{-3}$$

3 $$\kappa =\sqrt{\frac{2\times F^{2}\times I}{\varepsilon_{0}\times \varepsilon_{r}\times R\times T}}$$

where *u*_e_ is the electrophoretic mobility; η is the viscosity; ε_0_ = 8.854 × 10^–12^ F m^–1^; ε_r_ is the relative permittivity of the solvent; κ is the reciprocal Debye length; *r* is the radius of the colloidal particle; *F* is the Faraday constant; *I* is the ionic strength; *R* is the gas constant.

The surface charge density was estimated using the Ohshima–Healy–White equation (eq 4) for spherical particles, considering the ionic strength dependence of the ζ-potential instead of the surface potential (Ψ), which is a reasonable approximation for low- and moderately charged species.^24^

4 $$q_{s}=\frac{2{\times \varepsilon }_{r}\times \varepsilon_{0}\times \kappa \times R\times T}{F}\sinh\left(\frac{\Psi \times F}{2\times R\times T}\right){\left(1+\frac{2}{\kappa \times r\times \cos h^{2}\left(\Psi \times F/4\times R\times T\right)}+\frac{8\times \ln\left[\cosh\left(\Psi \times F/4\times R\times T\right)\right]}{{\left(\kappa \times r\right)}^{2}\sin h^{2}\left(\Psi \times F/2\times R\times T\right)}\right)}^{1/2}$$

where *q*_s_ is the surface charge density (C/m^2^ if the SI units are used; 1 C/m^2^ = 6.24 e/nm^2^).

The Fuchs function, eq 5, is key to determining the CCC value.

5 $$W=\frac{k_{r}}{k_{sl}}=\frac{{\left[{\left(\mathrm{dr}/\mathrm{dt}\right)}_{t\to 0}\right]}_{r}}{{\left(\mathrm{dr}/\mathrm{dt}\right)}_{t\to 0}}$$

where *k*_r_ and *k*_sl_ are the rate constants for rapid and slow coagulations, respectively.

To determine the coagulation rate, the size of the species in the working systems was monitored for 15 min. The coagulation rate value was estimated as the slope of the initial linear part of the size*–*time dependence using cumulant analysis of DLS data, i.e., mean hydrodynamic size *Z*_ave_. The dependence of *dZ*_aver_/*dtime* vs electrolyte concentration was treated by linear trends with respect to slow and rapid coagulation. The intersection point corresponds to CCC and was found algebraically.^21^

Vis-absorption spectra were obtained using a Hitachi U-2000 spectrophotometer and standard 1 cm quartz cuvettes. The absorbance of the working system with biological secretions was 0.2–0.3 and 0.10–0.15 at 310 and 405 nm, unless otherwise noted. According to the extinction coefficient from ref 32, this corresponds to (3–5) × 10^–5^ g/cm^3^. Also, the data on the extinction coefficient of TiO_2_ are presented in ref 33.

## 3 Results and Discussion

### 3.1 Colloidal Stability in Water, ACN, and DMSO

The TiO_2_ nanosample is colloidal unstable in distilled water at pH = 5.8, which is inherent to these systems. After a treatment of a two-phase system containing the 5 mg sample and 10 mL distilled water with ultrasound at a frequency of 50/60 Hz for 8 min, the sample precipitated, which was evident to the naked eye. Reference 34 discusses spectrophotometric monitoring of stability at different pH levels. The particle size and ζ-potential in aqueous systems of various pH values in ref 33 attribute the low colloidal stability to the weak ζ-potential and agglomeration of the particles at a bulk pH of 4–7. The size remained unchanged after one month of monitoring positive and negative particles at pH levels of 3 and 10, respectively.^33^ The effect of pH on the ζ-potential of anatase TiO_2_ is discussed in ref 35.

Around the point-of-zero charge (pH_PZC_, tabulated in ref 36), the surface energy of particles after the dispersion process in fluids is not stabilized by thermal motion energy and energy of interaction with water and is compensated by the reduction of surface area due to the sticking of nanoparticles together. The particle–particle attraction energy (*U*_A_) is expressed:^37^

6 $$U_{A}=-\frac{A^{*}}{6}\left[\frac{2}{s^{2}-4}+\frac{2}{s^{2}}+\ln\frac{s^{2}-4}{s^{2}}\right]$$

7 $$A^{*}={\left(A_{\mathrm{PP}}^{1/2}-A_{\mathrm{SS}}^{1/2}\right)}^{2}$$

where *A*_PP_ and *A*_SS_ are the Hamaker constants of particle–particle and solvent–solvent interactions, respectively; *s* = 2 + *h* × *r* ^–1^; *h* is the distance between the particles.

The Hamaker constant for TiO_2_ estimated as 25 zJ (10^–21^ J = zJ, anatase in water),^38^ 53.5 zJ (tetragonal rutile in water, the UV–IR undamped oscillator model),^39^ 53.5 zJ (rutile in water, the summation NinhamParsegian^39^), 60 zJ (rutile in water, the integral Kramers–Kronig^40^), 60 zJ (rutile in water, the Lifshitz theory using full spectral optical data obtained by vacuum ultraviolet and optical spectroscopy^40^), 39–100 zJ (rutile in water),^38^ 173 zJ (rutile in vacuum, the integral Kramers–Kronig^40^),153 zJ (rutile in vacuum, the summation NinhamParsegian^39^), 197 zJ (anatase in vacuum),^38^ and 110–310 zJ (rutile in vacuum).^38^ Compared to Hamaker constants of nanotube–nanotube (40–600 zJ^41^) and water–water (37–55 zJ^42^).

Altering bulk pH from the pH_PZC_ to the acidic or basic side, the contribution of the high Hamaker constant is balanced by electrostatic or charge stabilization. The energy of electrostatic repulsion (*U*_R_) is directly related to the value of the electric potential (Ψ) and the thickness of the electric double layer.^24^

8 $$U_{R}=64\times \pi \times \varepsilon_{r}\times \varepsilon_{0}{\left(\frac{R\times T}{F}\right)}^{2}\mathrm{tg}h^{2}\left(\frac{\Psi \times F}{4\times R\times T}\right)\frac{r\times \exp\left(-\kappa \times h\right)}{s}$$

According to DLVO theory (B. V. Derjaguin, L. D. Landau, E. J. W. Verwey, and J. T. G. Overbeek), the total potential energy is determined by the sum of *U*_R_, *U*_A_, and additional solvation contribution *U*_S_ = *K* × l×exp­(−*h*/*l*), where *K* and *l* are constants.^22^ In eq 8 for a particular system, the variables independent of the particles are the distance between the particles and the Debye length, but for nanotitania, the nature of the surface charge also depends on the dispersion medium, making a special contribution. The current concept of the nature of charge in aqueous media is formed, but for nonaqueous solvents, these aspects have been poorly elucidated.

The surface hydroxyl groups are chemically anchored to Ti^4+^ ions residing within the oxide crystal lattice. In an aqueous liquid, the surface electric potential of TiO_2_ undergoes substantial changes. Interaction of these surface groups with H^+^/OH^–^ ions initiates surface association–dissociation or interfacial adsorption–desorption process (≡Ti–OH + H^+^ ⇔ ≡Ti–OH_2_^+^) and deprotonation (≡Ti–OH + HO^–^ ⇔ ≡Ti–O^–^ + H_2_O).^43^ In this case, the H^+^/OH^–^ ions are potential-determining ions, as started by Bruyn^44^ or charge-determining ions initiated by Lyklema.^45^ Based on ref 46, water tends to adsorb in a molecular form on rutile (110) and anatase (100), whereas dissociative adsorption is favored on rutile (011) and anatase (001). The second layer forms an ordered water structure, adsorbed on the surface or on the terminal OH group via hydrogen bonds. The sign and magnitude of the surface electric charge depend on the ionized hydroxyl group, and the pH_PZC_ is characteristic of such surfaces. The surface charges generated lead to the formation of a double electric layer in electrolyte solutions, characterized by an electrokinetic potential with a specific isoelectric point.

Two solvents, i.e., acetonitrile and DMSO, were used in this study. The dispersions of nano-TiO_2_ were prepared by a 5-fold ultrasound treatment (50/60 Hz for 8 min) of a two-phase system containing 5 mg TiO_2_ and 10 mL of the solvent. The water in the ultrasonic bath was 25 °C. The systems were filtered through a paper filter (medium filtration rate, grade ST60, France). The absorption spectra (Figure S2) demonstrate better dispersibility in ACN. The behavior of acetonitrile consisted of a negative ζ-potential (−31 mV, Table 1) for the particles, imparting electrostatic stabilization in a low-polar environment, and the particles did not sediment during the month of observation. The created system was serially diluted and characterized by absorption spectrum (Figure S2) and hydrodynamic size (Table 2 and Figures S3–S9). The PdI of most of the system indicated that the sample has a narrow size distribution and may be considered monodisperse. Table 2 shows the DLS results of the cumulant analysis (*Z*_ave_ and PdI) and analysis for size distribution by intensity, volume, and particle number. An initial sample of TiO_2_ was characterized by a mean size of about 74 nm via transmission electron microscopy and 75 nm (by particle number) with ζ = −52 mV in water at pH = 6.65 by DLS.^5^ Origin of surface charge can be discussed based on current data of quasi-elastic neutron scattering, molecular dynamics (MD) simulation of a perfect passivized anatase slab,^47^ and density functional theory calculations for the clean TiO_2_(110).^48^ The first one detected several immobile ACN layers around nano-TiO_2_ particles. Based on MD simulation,^47^ the essential interaction between the nitrogen atom and the Ti atom is expected. This interaction originates from the dipole moment of ACN orienting away from the surface and producing a potential drop across the interface of ∼1.3 V.^49^ Density functional theory calculations show that pure ACN adopts three distinct adsorption modes: physical, chemisorption, and dissociated adsorption, where cleavage of the C–H bond produces a surface-bound cyanomethyl anion and a hydrogen atom.^48^ CH_2_CN group coordinates to a 5-fold-coordinated titanium site, and the hydrogen atom bonds to a bridging oxygen, producing a surface hydroxyl group. Acetonitrile’s unusually weak C–H bonds result from π-resonance stabilization.^50^ Reference 50 also includes Mulliken charges. The nanopowder used does not have an ideal structure, and standard Reichardt’s dye showed moisture in the solvent (Figure S1); thus, the surface can be hydroxylated through interaction with water. At the same time, the addition of water as a cosolvent to a 1:10 diluted initial system led to an increase in the ζ-potential: −(17 ± 2) and −(13 ± 4) mV at 20 and 40 vol % H_2_O and aggregation: an initial size of 740 nm (Figures S10 and S11).

**Table 1:** ζ-potential (mV) by the Hückel Model and Electrophoretic Mobility of Particles (μm × cm × V^–1^ × s^–1^) in ACN (0.001 mol % water) and DMSO (0.5 mol % water) with Different Dilutions of the Created Dispersions (*D*)

ACN			DMSO		
*D*	ζ	*u*_e_	*D*	ζ	*u*_e_
3:500	–(30 ± 1)	–(1.9 ± 0.1)	1:500	–(34 ± 1)	–(0.45 ± 0.05)
3:250	–(31 ± 1)	–(2.0 ± 0.1)	1:100	–(30 ± 1)	–(0.38 ± 0.05)
1:50	–(31 ± 1)	–(1.9 ± 0.1)	3:200	–(27 ± 1)	–(0.35 ± 0.05)
1:25	–(33 ± 1)	–(2.1 ± 0.1)	1:50	–(11 ± 4)	–(0.14 ± 0.09)
2:25	–(30 ± 2)	–(1.9 ± 0.1)	1:25	16 ± 3	0.21 ± 0.07
1:10	–(31 ± 3)	–(2.0 ± 0.2)	3:50	21 ± 2	0.19 ± 0.06
1:5	–(31 ± 4)	–(2.0 ± 0.3)	1:8	39 ± 1	0.52 ± 0.05
			2:5	57 ± 1	0.75 ± 0.05
			3:5	61 ± 1	0.79 ± 0.05
			1:1	65 ± 1	0.86 ± 0.05

**Table 2:** Hydrodynamic Particle Size and PdI in ACN with Different Dilutions of the Created Dispersion

		mean diameter, nm						
			by intensity			by volume		
*D*	PdI	*Z*_ave_	I	II	III	I	II	by number
3:500	0.356 ± 0.001	367 ± 1	503 ± 1	97 ± 1	4890 ± 100	735 ± 1	93 ± 1	95 ± 1
3:250	0.259 ± 0.001	194 ± 1	176 ± 1			176 ± 1		157 ± 1
1:50	0.114 ± 0.001	193 ± 1	207 ± 1			207 ± 1		159 ± 1
1:25	0.254 ± 0.001	185 ± 1	170 ± 1			167 ± 1		143 ± 1
2:25	0.140 ± 0.001	187 ± 1	212 ± 1			211 ± 1		136 ± 1
1:10	0.131 ± 0.001	184 ± 1	201 ± 1			200 ± 1		149 ± 1
1:5	0.162 ± 0.001	185 ± 1	200 ± 1	5120 ± 100		197 ± 1	5290 ± 100	135 ± 1

The systems in DMSO as a dispersion medium behaved quite differently. Specifically, a sign change of the ζ-potential occurred as the serial dilution varied (Table 1). Stable colloidal particles in DMSO exhibited a variable positive charge in systems with a higher portion of nanoparticles, while these particles maintained a fixed size. In contrast, negative unstable particles with varying sizes (Tables 1 and 3 and Figures S12–S21) were observed when their content was low. Around zero ζ-potential, the system was colloidally unstable. Considering the effect of moisture on surface properties, analysis of the system showed 0.5 mol % water in each DMSO system. For example, optimized structures and adsorption energies of DMSO on the pure and HO-preadsorbed CuO(111) surface, as well as on SBA-15 molecular sieves, are presented in refs 51,52,51,52 . The solvation of the Zn^2+^ in DMSO via X-ray absorption spectroscopy is given in ref 53.

**Table 3:** Gutmann Donor Number (DN), Mayer Acceptor Number (AN), Reichardt’s Parameter (*E*_T_(30)), and Kamlet–Taft Parameters of Water, ACN, and DMSO

solvent	DN^57^	AN^58^	*E*_T_(30)^59^	π*^59^	α^59^	β^59^
water	18.0	54.8	63.1	1.09	1.17	0.47
ACN	14.1	19.3	45.6	0.66	0.19	0.40
DMSO	29.8	19.3	45.1	1.00	0.00	0.76

The Mayer acceptor number, the Gutmann donor number, Reichardt’s parameter, and Kamlet–Taft parameters are collected in Table 4. A stronger donor ability of DMSO and a stronger acceptor ability of the H_2_O molecule imply affinity to the acidic and basic sites of the surface, respectively. Water as a cosolvent at 5 and 60 vol % led to a decrease in ζ-potential from 57 mV (2:5 diluted original) to 49 and 10 mV with *Z*_ave_ of 157.1 ± 0.1 and 277.3 ± 0.1 and PdI of 0.131 ± 0.001 and 0.013 ± 0.001, although the negative ζ-potential in the 1:100 diluted original remained with when added at 90 vol % (PdI = 0.636 ± 0.001, *Z*_ave_ = 452.7 ± 0.1, Figures S22–S24 and Tables S1 and S2).

**Table 4:** Hydrodynamic Size of Particle and PdI in DMSO at Different Dilutions of Created Dispersion

		mean diameter, nm					
			by intensity		by volume		
*D*	PdI	*Z*_ave_	I	II	I	II	by number
1:500	0.258 ± 0.001	294.9 ± 0.1	399.1 ± 0.1		456.4 ± 0.1		221.7 ± 0.1
1:100	0.267 ± 0.001	163.1 ± 0.1	148.7 ± 0.1		157.6 ± 0.1		137 ± 1
3:200	0.333 ± 0.001	544.8 ± 0.1	444.3 ± 0.1		433.9 ± 0.1		384.7 ± 0.1
1:50	0.335 ± 0.001	694.1 ± 0.1	618.3 ± 0.1	4920 ± 10	612.3 ± 0.1	5100 ± 10	484.5 ± 0.1
1:25	0.405 ± 0.001	232.8 ± 0.1	186.3 ± 0.1	5020 ± 10	204.8 ± 0.1	5180 ± 10	174.6 ± 0.1
3:50	0.320 ± 0.001	182.4 ± 0.1	158.4 ± 0.1		169.2 ± 0.1		148.5 ± 0.1
1:8	0.231 ± 0.001	148.2 ± 0.1	141.6 ± 0.1		151 ± 1		121.1 ± 0.1
2:5	0.152 ± 0.001	146.9 ± 0.1	166.5 ± 0.1		197.3 ± 0.1		110 ± 1
3:5	0.160 ± 0.001	143.9 ± 0.1	162.3 ± 0.1		158 ± 1		100 ± 1
1:1	0.108 ± 0.001	144.4 ± 0.1	161.5 ± 0.1		184.9 ± 0.1		125 ± 1

The findings tentatively suggest that in the DMSO systems, water creates negative charges and DMSO generates positive charges. The acid sites of the surface enhance the positive charge on the hydrogen atoms in DMSO. Following the analogy with ACN, DMSO is unlikely to form a dimsyl anion. DMSO enhances acidic properties. Water molecules preferentially should interact with surface oxygen because of its high Mayer acceptor number (Table 3), promoting adsorption. Simulation results suggest that water dissociation on TiO_2_(110) occurs due to defect electrons in the material rather than through direct contact between water adsorbates and bridging hydroxyl groups.^54^

The Ti–OH group plays a crucial role as a precursor to the reactive site in the aqueous degradation of methylene blue, MB, and DMSO suppressed this degradation.^55,56^ MB showed absorption maximum at 665, 670, 669, and 669 nm in DMSO (99.5 mol %) systems of TiO_2_-free, TiO_2_ (1:60), TiO_2_ (1:20), and TiO_2_ (1:3.5), see Figure S25a. The binding changed the ζ-potential from −(23 ± 1) to +(22 ± 5) mV for TiO_2_ (1:60), +(5 ± 2) to +(3 ± 2) mV for TiO_2_ (1:20), from +(50 ± 2) to +(49 ± 2) mV for TiO_2_ (2:7). MB absorption at maximum decreases over time; experimental graphs are given in Figure S26a. For TiO_2_ (1:20), coagulation was observed with and without dye using the DLS method: particles aggregate over time (Figure S26b). In TiO_2_ (1:60) and (1:20) systems with dye, the size was 270 ± 5 and 130 ± 2 nm (PdI of 0.29 and 0.10) and remained unchanged. Thus, in TiO_2_ (1:60) and (2:7) systems with dye, the particles were positive according to ELS, did not coagulate according to DLS, were in a bound state according to solvatochromism, and degradation was according to absorption kinetics. For ACN systems, MB had an absorption maximum at 664 nm for a 1:40 dilution TiO_2_ (Figure S25b) and its absorption did not decrease over time, see Figure S26a.

### 3.2 Colloidal Stability of Coated Nanospecies and Their Interactions with Electrolyte at pH = 5.8

The alternate mode affecting the stability of hydrophobic colloidal dispersion is steric hindrance involving surface coating to prevent the particles from coming into close proximity, at which attraction prevails. It was reported^60^ that colloidal surfactants at the nanoparticle synthesis stage affect not only aggregation but also the shape of nano-TiO_2_. In this way, biological secretions were injected into the two-phase aqueous system at pH 5.8 before ultrasound treatment. In addition to surface coating, lysozyme and bile salts generate surface charges and contribute to the formation of the double layer.

Aqueous dispersions of modified nano-TiO_2_ were prepared by a 5-fold ultrasound treatment (a frequency of 50/60 Hz for 8 min) of a two-phase system containing 5 mg of TiO_2_ and 10 mL of distilled water with 50 mg of LSZ (system **LSZ**), 50 mg of sodium cholate (system **NaCh**), or 50 mg of sodium deoxycholate (system **NaDCh**). The temperature of the water in the ultrasonic bath was 25 °C. The systems were filtered through a paper filter (medium filtration rate, grade ST60, France). The absorption spectra of the created systems (Figure S27) demonstrate better dispersibility with lysozyme. Repulsive forces due to thermal fluctuations of noncovalent modifiers may play a role in the stability.

The particle sizes of the 50-fold diluted system with LSZ were as follows *Z*_ave_ = 138 ± 2 nm, PdI = 0.17 ± 0.02, *d* (by intensity) = 142 ± 5 nm, *d* (by volume) = 164 ± 7 nm, *d* (by particle number) = 79 ± 2 nm. These particles are close in size to the state at pH 6.65 in water. LSZ radius of gyration is 1.49 nm according to small-angle X-ray scattering.^61^

The X-ray crystallography of lysozyme is available in ref 62. In distilled water with a pH of 5.8, lysozyme (isoelectric point at pH ≈ 11 ^63^) carries a formal positive charge. An effective modification of the nanoparticles by surface LSZ-coating results in the ζ = 29 ± 3 mV. According to ref 64, the LSZ aqueous system is stable up to 0.3 M NaCl, and the aggregate size reaches about 0.7 μm at 0.5 M NaCl. Nevertheless, the ζ-potential value of the enzyme-modified particles at the mentioned salt concentration remains positive, and the system does not demonstrate the pronounced salting out (Figures 2–4) that was observed for oxidized carbon nanotubes under similar conditions.^65^

The surface charge density of enzyme-modified particles in system **LSZ** was estimated by eq 4 from empirical values of ζ-potential at additions of 0.02–0.4 M NaCl and 2–80 mM CaCl_2_. Values obtained with both electrolytes are about 0.049 elemental charges per nm^2^ (7.8 × 10^–3^ C/m^2^).

Noncovalent modification by bile salts, i.e., NaCh (system **NaCh**) and NaDCh (system **NaDCh**), resulted in negative electrophoretic mobility −(1.86 ± 0.09) and −(1.74 ± 0.07) μm × cm × V^–1^ × c^–1^, which corresponds to ζ of −(36 ± 3) and −(33 ± 1) mV, respectively. The *ζ vs c*(NaCl) corresponds to negative surface charge density of bile-modified particles of 0.075 and 0.066 elemental charges per nm^2^ (1.2 × 10^–2^ and 1.06 × 10^–2^ C/m^2^) in systems **NaCh** and **NaDCh**. The particle size in system **NaCh** is defined as *Z*_ave_ = 145 ± 2 nm, PdI = 0.15 ± 0.01, *d* (by intensity) = 164 ± 6 nm, *d* (by volume) = 190 ± 8 nm, *d* (by particle number) = 85 ± 2 nm. The particle size in system **NaDCh** is characterized by *Z*_ave_ = 183 ± 3 nm, PdI = 0.25 ± 0.03, *d* (by intensity) = 166 ± 6 nm, *d* (by volume) = 175 ± 8 nm, *d* (by particle number) = 122 ± 5 nm. Note, the linear dimensions of bile monomers are ∼20 Å and ∼3.5 Å.^66^

The modified surfaces manifested themselves as a shift in the absorption spectrum of proflavin from 444 nm (ref 67) to 440, 439, and 441 nm in TiO_2_ dispersion with LSZ, NaCh, and NaDCh. This solvatochromic shift is a common practice when binding a sample and changing its micropolarity.

The electrolyte effect on the colloidal behavior of the modified nanoparticles was investigated in detail (Table 5). The coating by the biological secretions provides colloidal stability of the nanoparticles in distilled water: the particles retain their size and ζ-potential during a 2-week observation period when stored at 4 °C (Figure 1). At a certain concentration, NaCl destabilizes the systems **LSZ**, **NaCh**, and **NaDCh** and induces coagulation (Figures S28–S33). This concentration in system **LSZ**–TiO_2_ is lower than the free lysozyme begins to aggregate: see ref 64. In the system **LSZ** for NaCl and CaCl_2_, both CCC correspond to an ionic strength of 200–210 mmol/L and display *I* influence on the EDL compression (κ converges to 1.5 nm^–1^), which is due to a decrease in the diffusion of counterions into the bulk phase, which reduces the absolute value of zeta and particle repulsion.

**Figure 1:**
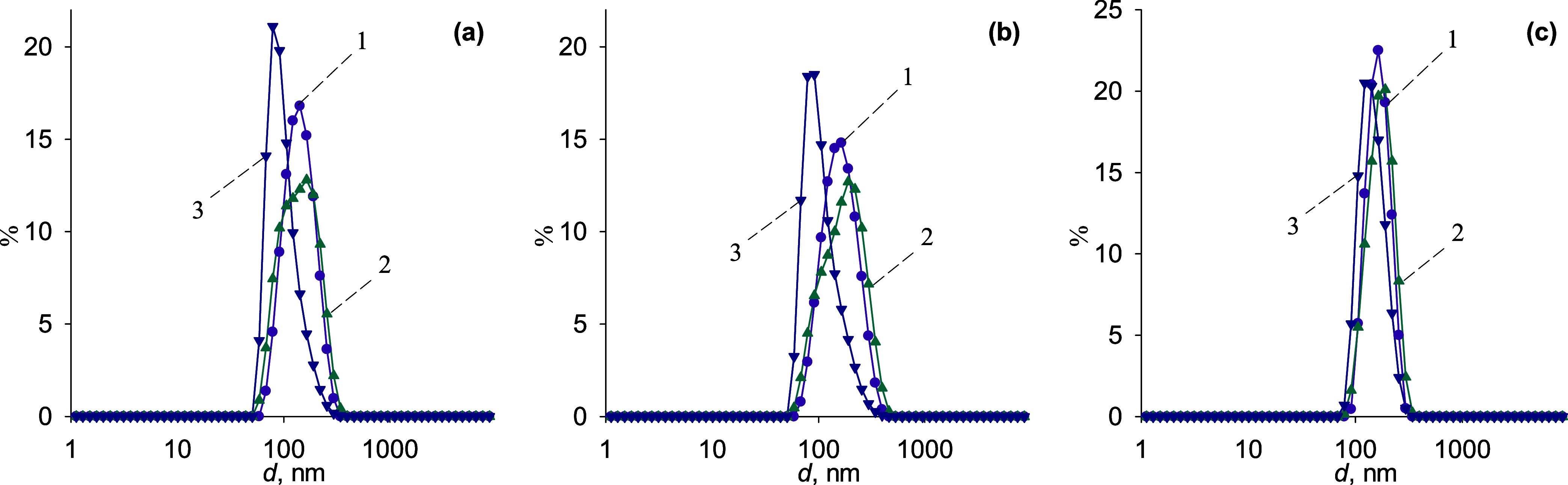
Size distribution by intensity (1), volume (2), and particle number (3) of aqueous dispersions of TiO_2_ with 50 mg LSZ (a), NaCh (b), and NaDCh (c).

**Table 5:** CCC Value for Nano-TiO_2_ Surface-Coated by LSZ, NaCh, and NaDCh

	CCC, mmol/L		
electrolyte	system LSZ	system NaCh	system NaDCh
NaCl	200 ± 10	90 ± 10	60 ± 10
CsI		60 ± 5	
CaCl_2_	70 ± 5	6.0 ± 0.5	6.5 ± 0.5
Sr(NO_3_)_2_		3.0 ± 0.5	
Na_2_SO_4_	12 ± 2		
NaCh	1.4 ± 0.2		
LSZ		0.40 ± 0.05 [mg/mL]	

Analyzing CCC­(NaCl) > CCC­(CsI), the stronger coagulation power of Cs^+^ is in line with their ionic radius and hydration radius. On the practical side, the results for Cs^+^ and Sr^2+^ (Figures 2–4) facilitate the search for effective scavengers for ^137^Cs and ^90^Sr as representatives of hazardous radionuclides. Poor overcharging does not result in stable systems, and they are removed from the liquid together with the nanoparticles.

For NaCl, i.e., a 1:1 electrolyte, coagulants are different ions, but the systems can be arranged in order of ionic strength, which corresponds to CCC. This value decreases in the modifier series of LSZ > NaCh > NaDCh, implying that lysozyme provides more favorable colloidal stabilization than the bile salts, despite the slightly lower *q*_*s*_. Bile salts have two sides of the steroidal backbone: hydrophilic on the concave α-side and hydrophobic on the convex β-side, and a negative charge at the end of the structure under physiological conditions.^68,69^ NaDCh is less hydrophilic, more hydrogen bond acidic, and slightly less polarizable than NaCh.^66^ LSZ with 129 residues in the native state has an 83% hydrophobic surface.^70^ The surface active centers of TiO_2_ can interact with the functional group of the biological secretions through noncovalent electrostatic interactions and hydrogen bonds, thus exposing the hydrophobic part of the biological secretions to the interface. Some surface hydrophobization was recently observed when LSZ-coated oxidized carbon nanotubes, OCN,^65^ leading to a lower CCC­(NaCl) than in “enzyme-free OCN” and “LSZ–TiO_2_” systems. Comparing the colloidal stability of nanotubes and nano-TiO_2_ coated with LSZ, the latter have a higher CCC, which indicates lower hydrophobicity of the systems obtained here. In contrast to the ζ-potential behavior observed in Figure 2, the charge inversion of nanoparticles in the “LSZ–OCN” system occurred at NaCl concentration corresponding to the aggregation of free lysozyme (i.e., > 0.3 mol/L NaCl).^64^ Remaining positive zeta value in “LSZ–TiO_2_” system with >0.3 mol/L NaCl (Figure 2) may be an attribute of lysozyme binding more strongly to nanotitania than to OCN. If so, the charge inversion in system **LSZ** with >9 mmol/L NaCh and >14 mmol/L Na_2_SO_4_ (by the way, as well as in systems **NaCh** and **NaDCh** over 0.1 mol/L Ca^2+^ and 0.05 mol/L Sr^2+^, see below) indicates the adsorption of the counterions. The bile anion and LSZ as counterions leading to overcharging colloidally stabilize LSZ–TiO_2_ and NaCh–TiO_2_, respectively (Figures 2 and 3, the stability interval: the *dZ*_ave_/*dt* → 0 after the plateau of rapid coagulation).

**Figure 2:**
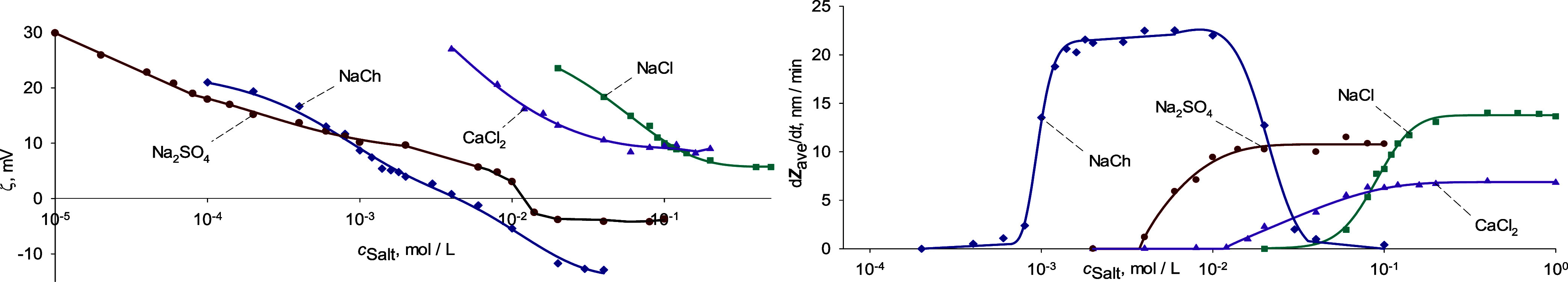
Dependences of the ζ-potential (on the left) and the rates of size increasing (on the right) vs concentrations of NaCl (square), CaCl_2_ (triangle), Na_2_SO_4_ (circle), and NaCh (diamond) in aqueous dispersion of TiO_2_ with LSZ.

**Figure 3:**
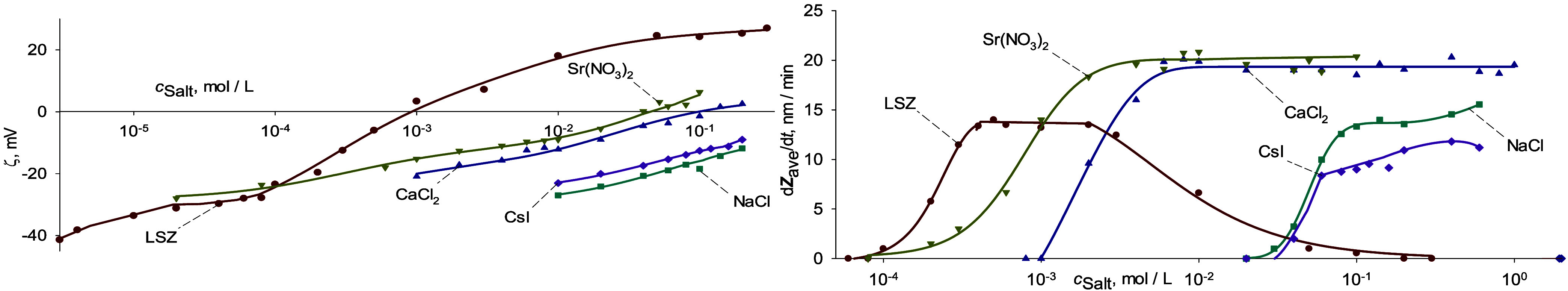
Dependences of the ζ-potential (on the left) and the rates of size increasing (on the right) vs concentrations of NaCl (square), CsI (diamond), CaCl_2_ (triangle), Sr­(NO_3_)_2_ (triangle down), and LSZ (circle) in aqueous dispersion of TiO_2_ with 5 mg/mL NaCh.

The ratio of CCC for mono and divalent inorganic counterions in systems **LSZ** and **NaCh** equals 15–17, which is smaller than the predicted effect of coagulant charge on hydrophobic nanoparticle by the classical DLVO theory. It is predicted that to maintain colloidal stability, the thickness of the anchored modifier layer of a completely covered particle must be >5 nm,^71^ which a monolayer of used modifiers may not provide. The divalent inorganic counterions at a certain concentration (Figures 2–4) cause a reversal in the electrophoretic movement of particles. In aqueous NaDCh, the calcium ion is 1:1 coordinated to oxygen atoms of the carboxylate group and water molecule by means of ion–ion and ion–dipole interactions.^72^

**Figure 4:**
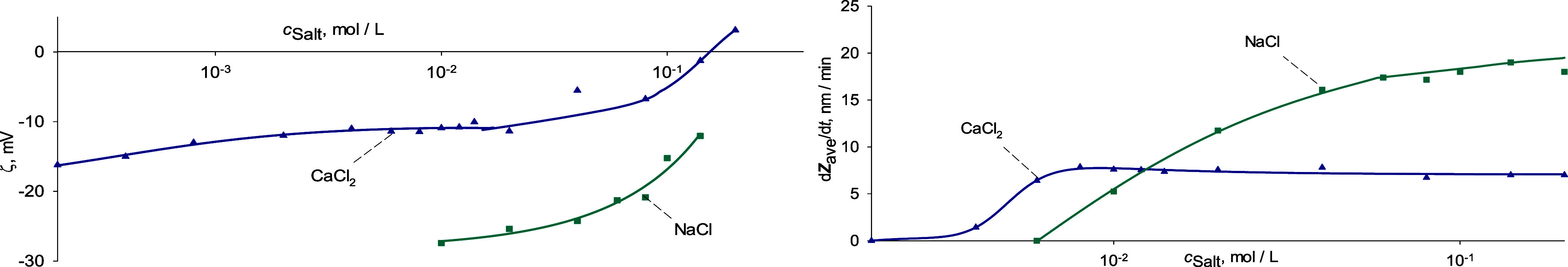
Dependences of the ζ-potential (on the left) and the rates of size increasing (on the right) vs concentrations of NaCl (square) and CaCl_2_ (triangle) in aqueous dispersion of TiO_2_ with 5 mg/mL NaDCh.

## 4 Conclusions

The search for a functional microenvironment for hydrophobic nanodispersions in liquids is an outstanding task for understanding surface effects. The data presented here are fundamental in nature and do not concern specific applications of the empirical data obtained, to keep the imagination free to roam for further research and practical applications. The primary intended audience is colloid chemists, biologists, and medical professionals seeking a fresh perspective on nanotitania. Chemists specializing in the sol–gel process required for preparing nano-TiO_2_, surface and bulk functionalization/modification methods, photocatalysts, food and drug colorants, adsorbents, and cosmetics should integrate these findings into the development strategy for high-performance, safe target products.

The colloidal behavior of the systems was evaluated by measuring the nanoparticles’ hydrodynamic size and ζ-potential using dynamic and electrophoretic light scattering. The nano-TiO_2_ is colloidal unstable in water around the isoelectric point. Distributing charged species in the system (electrostatic stabilization) tends to minimize the surface energy and affects the surface charge and mutual repulsion. Adsorption of solvents and modifiers is involved in the creation of dispersed systems. For the TiO_2_ sample kindly supplied by a colleague, solvents, ACN and DMSO, were chosen based on donor–acceptor properties. Modifiers, lysozyme, and bile salts, in aqueous systems, were selected based on the scope of TiO_2_ application in vivo. All colloidally stable systems created did not precipitate during the month of observation, compared to the separation of the deionized aqueous dispersion at pH = 5.8 within 5 min. Colloidal stability was controlled and manipulated in electrolyte solutions. As a result, a set of experimental data was provided for the application and fate of these nanosystems, as well as an understanding of surface effects.

The main difference between the influence of ACN and DMSO on dispersion is the different direction of electrophoretic motion of nanoparticles, because of negative and positive ζ-potentials. Common to these systems is that water weakens the ζ-potential. The working hypothesis for the origin of the surface charge is the coordination of the solvent to the acid center of the nanoparticles. As shown in the reference MD, the α-hydrogen of ACN can bind to the surface oxygen. In this case, the positive charge of the DMSO is retained in the presence of moisture. If this is only an assumption, then the zeta values are regarded as useful empirical information. In DMSO systems, methylene blue was in a bound state and degraded.

Modification with lysozyme and bile salts also results in different directions of migration of colloidal particles. At the same time, repeated cross-injection of secretions at certain concentrations changes the direction. Modification with lysozyme and bile salts does not provide stability of the system under the action of the electrolytes. The coagulating effect was quantified, resulting in the critical coagulation concentrations. Proflavine was bound to the modified surfaces.

Colloidal stability in relation to sodium chloride is higher in nano-TiO_2_ coated with lysozyme than in nanotubes. Modification with cholate and deoxycholate also results in different stabilities. This indicates that the interaction of the secretions with the functional sites of the nanoparticles controls the orientation of the modifier.

## Supplementary Material



## Notes

Absorption spectra of the discussed system; size distribution by intensity, volume, and particle number of the discussed system; the ζ-potential and electrophoretic mobility of particles; hydrodynamic size of particle and polydispersity index of the discussed system; Absorption spectra of MB in ACN and DMSO systems; absorption and *Z*_ave_ vs time dependences for MB in ACN and DMSO systems; kinetics of particle aggregation at different salt concentrations. (PDF)

## References

[1] Kumaravel V., Nair K. M., Mathew S., Bartlett J., Kennedy J. E., Manning H. G., Whelan B. J., Leyland N. S., Pillai S. C. (2021). Antimicrobial
TiO2 nanocomposite coatings for surfaces, dental and orthopaedic implants. Chem. Eng. J..

[2] Haghighi F. H., Mercurio M., Cerra S., Salamone T. A., Bianymotlagh R., Palocci C., Spica V. R., Fratoddi I. (2023). Surface modification
of TiO 2 nanoparticles with organic molecules and their biological
applications. J. Mater. Chem. B.

[3] Berardinelli, A. ; Parisi, F. TiO_2_ in the food industry and cosmetics. In Titanium Dioxide (TiO_2_) and Its Applications; Parrino, F. ; Palmisano, L. , Eds.; Elsevier, 2021; Chapter 11, pp 353–371.

[4] Guo Q., Zhou C., Ma Z., Yang X. (2019). Fundamentals of TiO2
Photocatalysis: Concepts, Mechanisms, and Challenges. Adv. Mater..

[5] Usenko E., Glamazda A., Valeev V., Svidzerska A., Laguta A., Petrushenko S., Karachevtsev V. (2022). Effect of
TiO2 nanoparticles on the thermal stability of native DNA under UV
irradiation. Appl. Phys. A.

[6] Scanlon D. O., Dunnill C. W., Buckeridge J., Shevlin S. A., Logsdail A. J., Woodley S. M., Catlow C. R. A., Powell M. J., Palgrave R. G., Parkin I. P. (2013). Band
alignment of rutile and anatase TiO2. Nat. Mater..

[7] Morales-García Á., Macià
Escatllar A., Illas F., Bromley S. T. (2019). Understanding
the interplay between size, morphology and energy gap in photoactive
TiO_2_ nanoparticles. Nanoscale.

[8] Abdullah M., Low G. K. C., Matthews R. W. (1990). Effects of common inorganic anions
on rates of photocatalytic oxidation of organic carbon over illuminated
titanium dioxide. J. Phys. Chem. A.

[9] Chang H., Wang Q., Meng X., Chen X., Deng Y., Li L., Yang Y., Song G., Jia H. (2022). Effect of titanium
dioxide nanoparticles on mammalian cell cycle in vitro: a systematic
review and meta-analysis. Chem. Res. Toxicol..

[10] Dedman C. J., King A. M., Christie-Oleza J. A., Davies G.-L. (2021). Environmentally
relevant concentrations of titanium dioxide nanoparticles pose negligible
risk to marine microbes. Environ. Sci.: Nano.

[11] Kim M., Goerzen D., Jena P. V., Zeng E., Pasquali M., Meidl R. A., Heller D. A. (2024). Human and
environmental safety of
carbon nanotubes across their life cycle. Nat.
Rev. Mater..

[12] Galletti A., Seo S., Joo S. H., Su C., Blackwelder P. (2016). Effects of
titanium dioxide nanoparticles derived from consumer products on the
marine diatom Thalassiosira pseudonana. Environ.
Sci. Pollut. Res..

[13] Chatterjee S., Sil P. C. (2024). Mechanistic Insights into Toxicity
of Titanium Dioxide
Nanoparticles at the Micro- and Macro-levels. Chem. Res. Toxicol..

[14] Li S.-Q., Zhu R.-R., Zhu H., Xue M., Sun X.-Y., Yao S.-D., Wang S.-L. (2008). Nanotoxicity of
TiO2 nanoparticles
to erythrocyte in vitro. Food Chem. Toxicol..

[15] Usenko E., Glamazda A., Svidzerska A., Valeev V., Laguta A., Petrushenko S., Karachevtsev V. (2023). DNA:TiO2 nanoparticle nanoassemblies:
effect of temperature and nanoparticle concentration on aggregation. J. Nanopart. Res..

[16] Chifor E., Bordeianu I., Anastasescu C., Calderon-Moreno J. M., Bratan V., Eftemie D.-I., Anastasescu M., Preda S., Plavan G., Pelinescu D. (2022). Bioactive Coatings Based on Nanostructured TiO2Modified with Noble
Metal Nanoparticles and Lysozyme for Ti Dental Implants. Nanomaterials.

[17] Zhao F. H., Chen Y. M., Hu Y., Lu X. G., Xiong S. B., Wu B. Y., Guo Y. Q., Huang P., Yang B. C. (2020). Conformation
changes of albumin and lysozyme on electrospun TiO2 nanofibers and
its effects on MSC behaviors. Colloids Surf.,
B.

[18] Liu Y., Jin Z., Meng H., Zhang X. (2018). Study on the enhanced adsorption
properties of lysozyme on polyacrylic acid modified TiO2 nano-adsorbents. Mater. Res. Express.

[19] Benbow N. L., Rozenberga L., McQuillan A. J., Krasowska M., Beattie D. A. (2021). ATR FTIR Study of
the Interaction of TiO2 Nanoparticle
Films with β-Lactoglobulin and Bile Salts. Langmuir.

[20] Tadros, T. Electrostatic and Steric Stabilization of Colloidal Dispersions, 2012.

[21] Laguta A. (2025). Determination
of the Critical Concentration of Rapid Coagulation by the Dynamic
Light Scattering Technique. J. Chem. Educ..

[22] Laguta A. N., Mchedlov-Petrossyan N., Kovalenko S., Voloshina T., Haidar V., Filatov D. Y., Trostyanko P., Karbivski V., Bogatyrenko S., Xu L., Prezhdo O. V. (2022). Stability
of aqueous suspensions of COOH-decorated carbon nanotubes to organic
solvents, esterification, and decarboxylation. J. Phys. Chem. Lett..

[23] Laguta A. N., Mchedlov-Petrossyan N.
O., Bogatyrenko S. I., Kovalenko S. M., Bunyatyan N. D., Trostianko P. V., Karbivskii V. L., Filatov D. Y. (2022). Interaction of aqueous suspensions
of single-walled oxidized carbon nanotubes with inorganic and organic
electrolytes. J. Mol. Liq..

[24] Kryshtal A., Mchedlov-Petrossyan N., Laguta A., Kriklya N., Kruk A., Osawa E. (2021). Primary detonation
nanodiamond particles: Their core-shell structure
and the behavior in organo-hydrosols. Colloids
Surf. A.

[25] Mchedlov-Petrossyan N. O., Kriklya N. N., Laguta A. N., O̅sawa E. (2022). Stability
of detonation nanodiamond colloid with respect to inorganic electrolytes
and anionic surfactants and solvation of the particles surface in
DMSO–H2O organo-hydrosols. Liquids.

[26] Dukhopelnikov E., Blyzniuk I., Bereznyak E., Gladkovskaya N., Vakula A., Sova K., Laguta A., Kaman O., Kubíčková L., Pashchenko M. (2025). Proflavine
binding to La-Sr perovskite Manganite nanoparticles at temperatures
above and below the Curie temperature. Colloids
Surf. A.

[27] Zetasizer Nano. User Manual, Man0485, Issue 1.1, 2013.

[28] Stetefeld J., McKenna S. A., Patel T. R. (2016). Dynamic
light scattering: a practical
guide and applications in biomedical sciences. Biophys. Rev..

[29] Folkerts, M. M. ; Yablonovich, A. Dynamic Light Scattering Malvern; 2010.

[30] Bhattacharjee S. (2016). DLS and zeta
potential–what they are and what they are not?. J. Controlled Release.

[31] Delgado Á. V., González-Caballero F., Hunter R., Koopal L., Lyklema J. (2007). Measurement and interpretation of
electrokinetic phenomena. J. Colloid Interface
Sci..

[32] Cabrera M. I., Alfano O. M., Cassano A. E. (1996). Absorption and scattering
coefficients
of titanium dioxide particulate suspensions in water. J. Phys. Chem. A.

[33] Joo N.-Y., Lee J., Kim S. J., Park H. M., Yun W. S., Yoon M., Song N. W. (2013). Preparation
of an aqueous suspension of stabilized
TiO2 nanoparticles in primary particle form. J. Nanosci. Nanotechnol..

[34] Martyanov I., Savinov E., Klabunde K. (2003). Influence of solution composition
and ultrasonic treatment on optical spectra of TiO2 aqueous suspensions. J. Colloid Interface Sci..

[35] Leong Y.-K., Ong B. (2003). Critical zeta potential
and the Hamaker constant of oxides in water. Powder Technol..

[36] Kosmulski M. (2016). Isoelectric
points and points of zero charge of metal (hydr)­oxides: 50years after
Parks’ review. Adv. Colloid Interface
Sci..

[37] Kovalchuk N., Starov V. (2012). Aggregation in colloidal suspensions: Effect of colloidal
forces and hydrodynamic interactions. Adv. Colloid
Interface Sci..

[38] Visser J. (1972). On Hamaker
constants: A comparison between Hamaker constants and Lifshitz-van
der Waals constants. Adv. Colloid Interface
Sci..

[39] Bergström L. (1997). Hamaker constants
of inorganic materials. Adv. Colloid Interface
Sci..

[40] Ackler H. D., French R. H., Chiang Y.-M. (1996). Comparisons of Hamaker Constants
for Ceramic Systems with Intervening Vacuum or Water: From Force Laws
and Physical Properties. J. Colloid Interface
Sci..

[41] Maeno Y., Ishikawa A., Nakayama Y. (2010). Adhesive behavior of single carbon
nanotubes. Appl. Phys. Express..

[42] Israelachvili, J. N. Intermolecular and Surface Forces; Academic Press, 2011.

[43] Spasojević L., Ivanišević I., Sikirić M. D. (2025). Stability
and application of TiO 2 nanomaterials in aqueous suspensions: a review. RSC Adv..

[44] Parks G. A., Bruyn P. L. (1962). The Zero Point of Charge of Oxides. J. Phys. Chem. A.

[45] Lyklema J. (1991). Nomenclature,
symbols, definitions and measurements for electrified interfaces in
aqueous dispersions of solids (Recommendations 1991). Pure Appl. Chem..

[46] Maleki F., Di Liberto G., Pacchioni G. (2023). pH- and Facet-Dependent Surface Chemistry
of TiO2 in Aqueous Environment from First Principles. ACS Appl. Mater. Interfaces.

[47] Vaissier V., Sakai V. G., Li X., Cabral J. T., Nelson J., Barnes P. R. (2016). How mobile are dye
adsorbates and acetonitrile molecules
on the surface of TiO2 nanoparticles? A quasi-elastic neutron scattering
study. Sci. Rep..

[48] Zou X.-J., Ding K. N., Zhang Y. F., Li J. Q. (2011). A DFT study of acetonitrile
adsorption and decomposition on the TiO2 (110) surface. Int. J. Quantum Chem..

[49] Blazhynska M. M., Stepaniuk D. S., Koverga V., Kyrychenko A., Idrissi A., Kalugin O. N. (2021). Structure
and dynamics of TiO2-anchored
D205 dye in ionic liquids and acetonitrile. J. Mol. Liq..

[50] Goebbert D. J., Velarde L., Khuseynov D., Sanov A. (2010). C–H Bond Dissociation
Energy of Malononitrile. J. Phys. Chem. Lett..

[51] Xia H., Meng X., Jiang X., Lu L., Wang Y. (2023). Theoretical
Investigation on the Catalytic Effect and Mechanism of Pure and Cu–Doped
SBA–15 Molecular Sieves on the Decomposition of Dimethyl Sulfoxide. Processes.

[52] Wang Y.-Q., Meng X.-L., Xia H.-H., Su J.-Z., Lu L.-L., Yu W.-C. (2023). Investigation on the Catalytic Cracking Mechanism of CuO on Dimethyl
Sulfoxide (C2H6OS) and Surface Modification Effects: Insights from
Density Functional Theory Calculations. Processes.

[53] Zitolo A., D’Angelo P. (2010). X-ray absorption
spectroscopy study of the solvation
structure of zinc­(II) in dimethyl sulfoxide solution. Chem. Phys. Lett..

[54] Petrik N. G., Baer M. D., Mundy C. J., Kimmel G. A. (2022). Mixed Molecular
and Dissociative Water Adsorption on Hydroxylated TiO2(110): An Infrared
Spectroscopy and Ab Initio Molecular Dynamics Study. J. Phys. Chem. C.

[55] Du P., Bueno-López A., Verbaas M., Almeida A. R., Makkee M., Moulijn J. A., Mul G. (2008). The effect of surface OH-population
on the photocatalytic activity of rare earth-doped P25-TiO2 in methylene
blue degradation. J. Catal..

[56] Shimizu N., Ogino C., Dadjour M. F., Murata T. (2007). Sonocatalytic degradation
of methylene blue with TiO2 pellets in water. Ultrason. Sonochem..

[57] Gutmann V. (1976). Empirical
parameters for donor and acceptor properties of solvents. Electrochim. Acta.

[58] Mayer U., Gutmann V., Gerger W. (1975). The acceptor
number—A quantitative
empirical parameter for the electrophilic properties of solvents. Monatsh. Chem..

[59] Reichardt, C. ; Welton, T. Solvents and Solvent Effects in Organic Chemistry; John Wiley & Sons, 2011.

[60] Loosli F., Stoll S. (2017). Effect of surfactants, pH and water hardness on the surface properties
and agglomeration behavior of engineered TiO 2 nanoparticles. Environ. Sci.: Nano.

[61] Nikfarjam S., Ghorbani M., Adhikari S., Karlsson A., Jouravleva E., Woehl T., Anisimov M. (2019). Irreversible
nature of mesoscopic
aggregates in lysozyme solutions. Colloid J..

[62] Diamond R. (1974). Real-space
refinement of the structure of hen egg-white lysozyme. J. Mol. Biol..

[63] Ibáñez R., Almécija M. C., Guadix A., Guadix E. M. (2007). Dynamics of the
ceramic ultrafiltration of model proteins with different isoelectric
point: comparison of β-lactoglobulin and lysozyme. Sep. Purif. Technol..

[64] Poznański J., Szymanski J., Basinska T., Słomkowski S., Zielenkiewicz W. (2005). Aggregation
of aqueous lysozyme solutions followed
by dynamic light scattering and 1H NMR spectroscopy. J. Mol. Liq..

[65] Laguta A. (2025). Colloidal
behavior of oxidized and lysozyme-coated single-walled carbon nanotubes.
Analysis via dynamic and electrophoretic light scattering. V. N. Karazin Kharkiv Natl. Univ. Bull. Chem. Ser..

[66] Monte M. J., Marin J. J., Antelo A., Vazquez-Tato J. (2009). Bile acids:
chemistry, physiology, and pathophysiology. World J. Gastroenterol..

[67] Albert A. (1965). 864. Acridine
syntheses and reactions. Part VI. A new dehalogenation of 9-chloroacridine
and its derivatives. Further acridine ionisation constants and ultraviolet
spectra. J. Chem. Soc..

[68] di
Gregorio M. C., Cautela J., Galantini L. (2021). Physiology
and Physical Chemistry of Bile Acids. Int. J.
Mol. Sci..

[69] Režen T., Rozman D., Kovács T., Kovács P., Sipos A., Bai P., Mikó E. (2022). The role of
bile acids in carcinogenesis. Cell. Mol. Life
Sci..

[70] Cao J., Pham D., Tonge L., Nicolau D. (2002). Predicting surface
properties of proteins on the Connolly molecular surface. Smart Mater. Struct..

[71] Tadros, T. General Principles of Colloid Stability and the Role of Surface Forces. In Colloid Stability; Wiley, 2010; pp 1–22.

[72] D’Archivio A.
A., Galantini L., Gavuzzo E., Giglio E., Mazza F. (1997). Calcium Ion
Binding to Bile Salts. Langmuir.

