# Supplementary material for: Versatility of TiO2 Nanoparticles Surfaces in ACN, DMSO, and Aqueous Natural Secretions: Colloidal Behavior
Source: ACS Omega. 2026 May 18;11(21):31702–11. doi: 10.1021/acsomega.6c02670 (PMC13234789; doi:10.1021/acsomega.6c02670)
Supplement: Supplementary file 1 [file ao6c02670_si_001.pdf]

Supplementary Material

Versatility of TiO_2 nanoparticles surfaces in ACN, DMSO, and aqueous natural secretions: colloidal behavior

Anna Laguta*[1,2]

[1] University of Chemistry and Technology Prague, Technická 5, 166 28 Prague 6, Czech Republic
[2] V. N. Karazin Kharkiv National University, Svoboda Square 4, Kharkiv 61022, Ukraine

***laguta@karazin.ua**

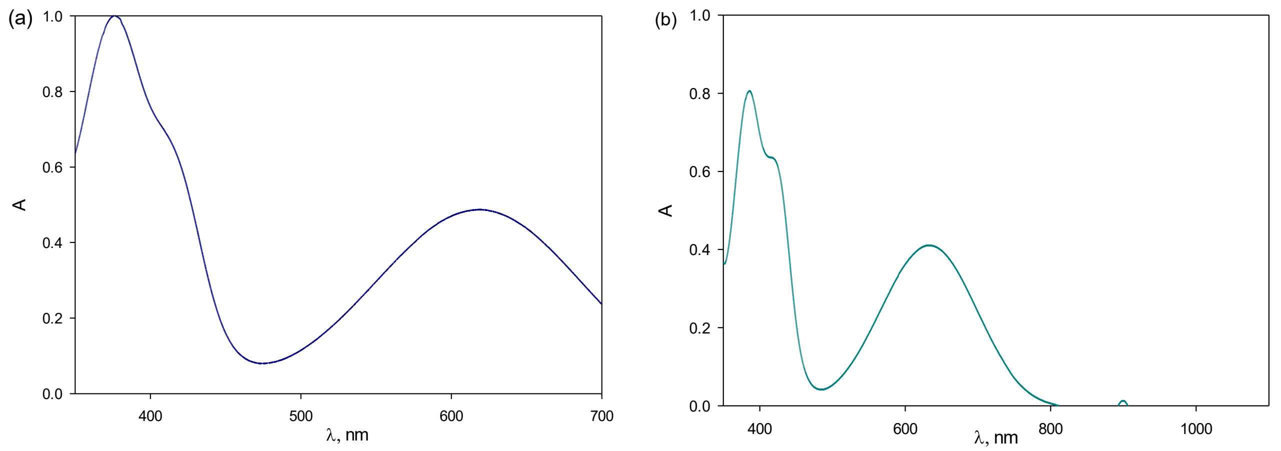

Fig. S1. Absorption spectra of Reichardt's standard dye in DMSO and acetonitrile with moisture of water (context: DMSO and acetonitrile contained 0.5 and 0.001 mol.% water, respectively, as estimated by Reichardt's standard dye-based solvent polarity (λ_{max} = 633 and 618 nm, respectively).

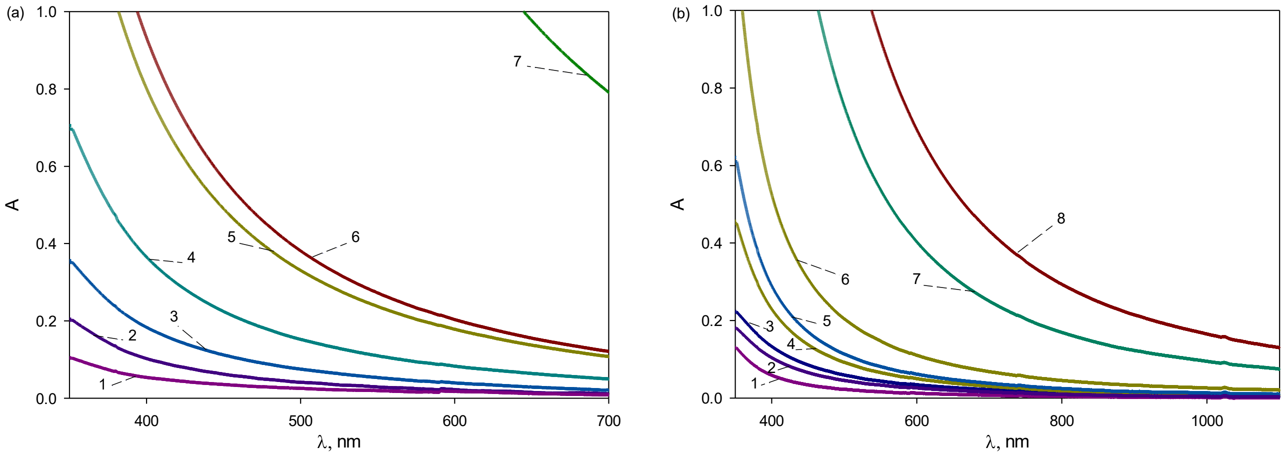

Fig. S2. Absorption spectra of ACN (a) and (b) DMSO working TiO_2 suspensions. The stock system dilution was 3:500 (1), 3:250 (2), 1:50 (3), 1:25 (4), 2:25 (5), 1:10 (6), and 1:5 (7) for ACN (a) and 1:100 (1), 3:200 (2), 1:50 (3), 1:25 (4), 3:50 (5), 1:8 (6), 2:5 (7), and 3:5 (8) for DMSO (b).

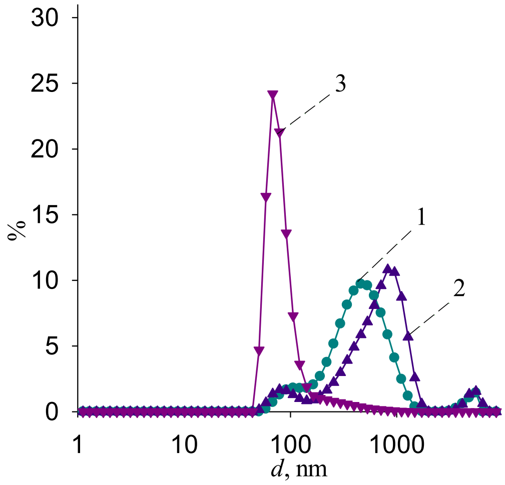

Fig. S3. Size distribution by intensity (1), volume (2), and particle number (3) of a 3:500 diluted initial system in ACN.

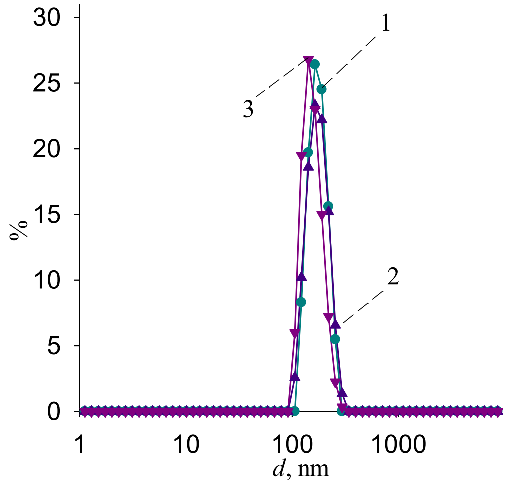

Fig. S4. Size distribution by intensity (1), volume (2), and particle number (3) of a 3:250 diluted initial system in ACN.

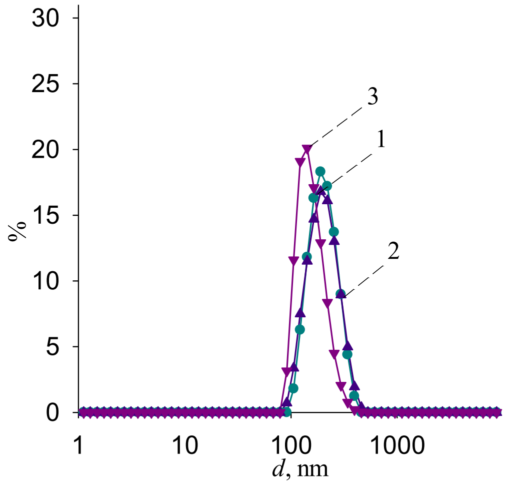

Fig. S5. Size distribution by intensity (1), volume (2), and particle number (3) of a 1:50 diluted initial system in ACN.

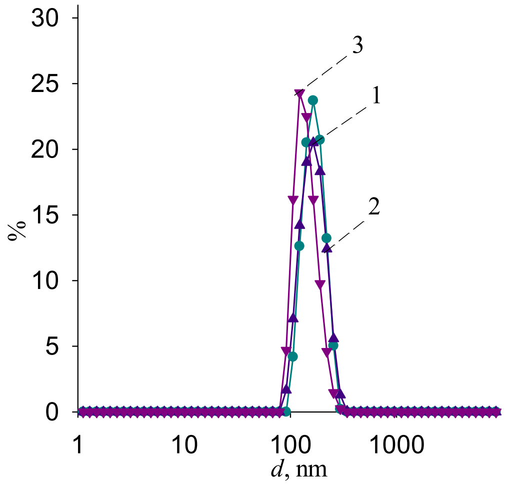

Fig. S6. Size distribution by intensity (1), volume (2), and particle number (3) of a 1:25 diluted initial system in ACN.

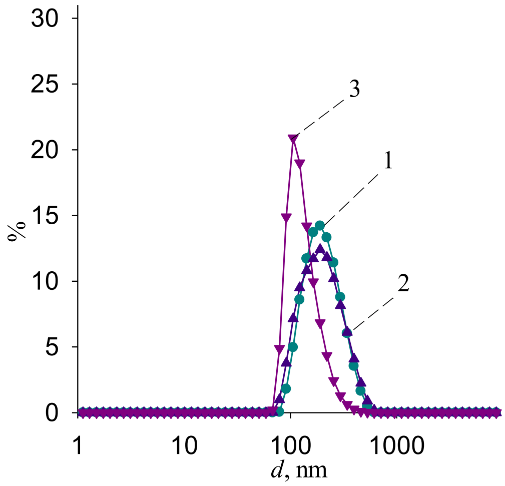

Fig. S7. Size distribution by intensity (1), volume (2), and particle number (3) of a 2:25 diluted initial system in ACN.

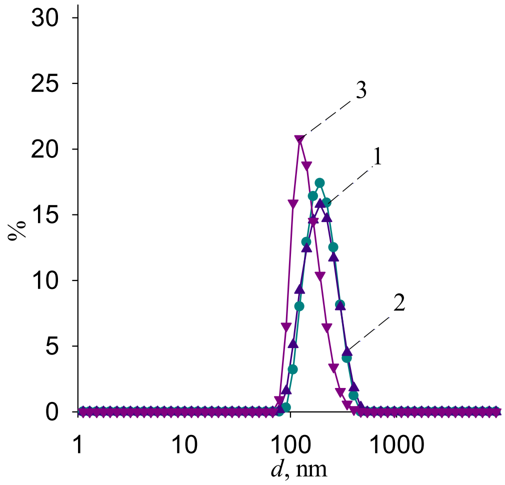

Fig. S8. Size distribution by intensity (1), volume (2), and particle number (3) of a 1:10 diluted initial system in ACN.

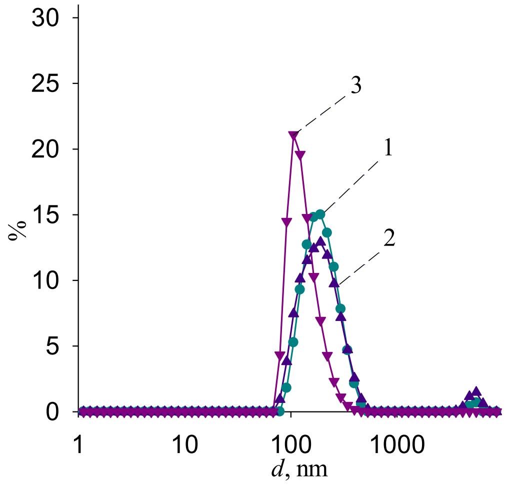

Fig. S9. Size distribution by intensity (1), volume (2), and particle number (3) of a 1:5 diluted initial system in ACN.

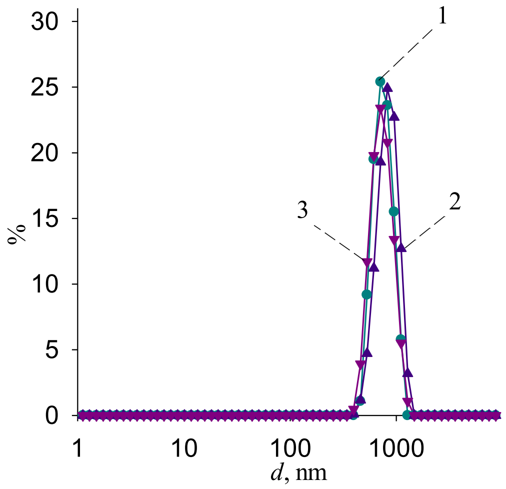

Fig. S10. Size distribution by intensity (1), volume (2), and particle number (3) of a 1:10 diluted initial system in ACN with 20 v% H_2O at initial time.

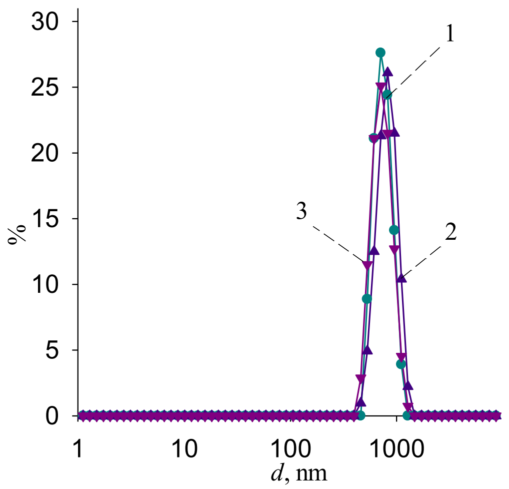

Fig. S11. Size distribution by intensity (1), volume (2), and particle number (3) of a 1:10 diluted initial system in ACN with 40 v% H_2O at initial time.

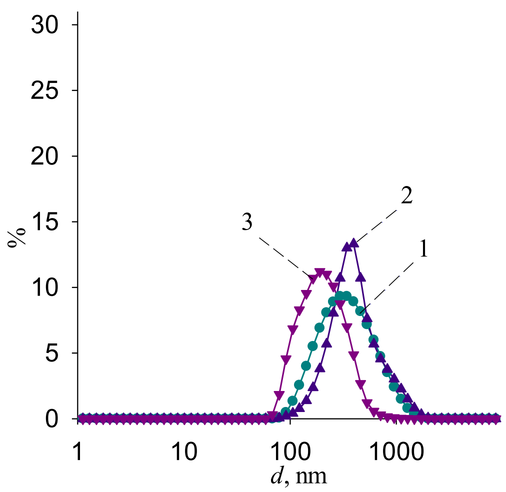

Fig. S12. Size distribution by intensity (1), volume (2), and particle number (3) of a 1:500 diluted initial system in DMSO.

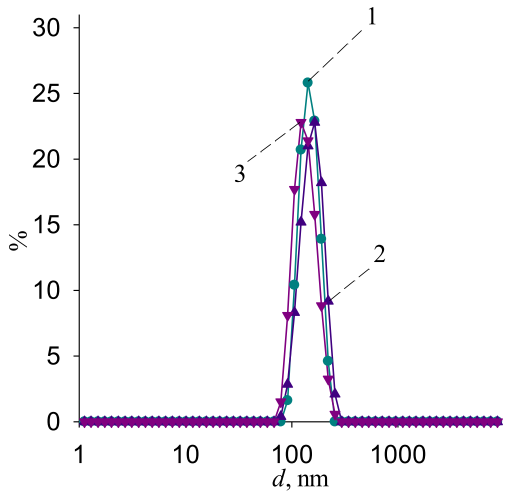

Fig. S13. Size distribution by intensity (1), volume (2), and particle number (3) of a 1:100 diluted initial system in DMSO.

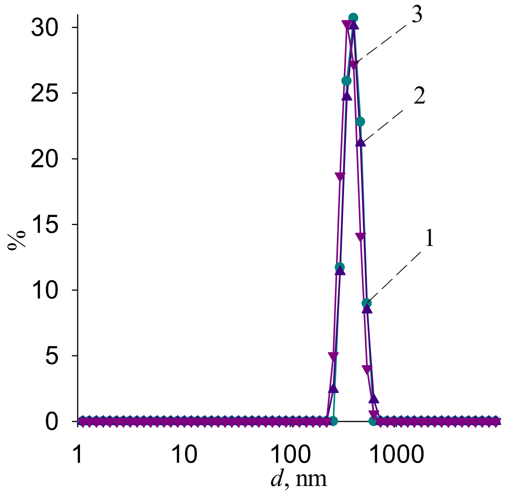

Fig. S14. Size distribution by intensity (1), volume (2), and particle number (3) of a 3:200 diluted initial system in DMSO.

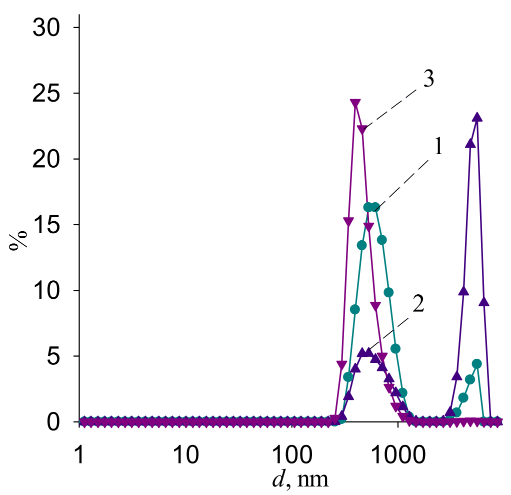

Fig. S15. Size distribution by intensity (1), volume (2), and particle number (3) of a 1:50 diluted initial system in DMSO.

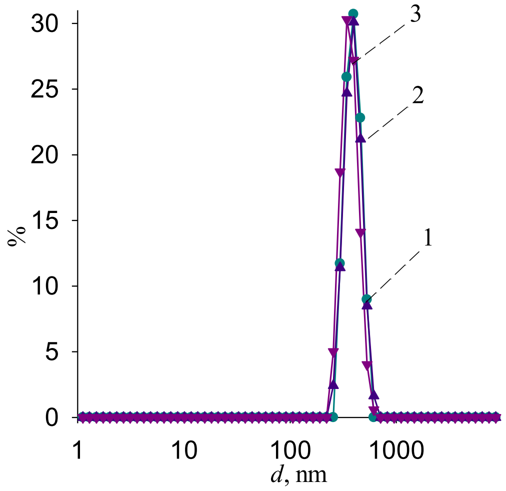

Fig. S16. Size distribution by intensity (1), volume (2), and particle number (3) of a 1:25 diluted initial system in DMSO.

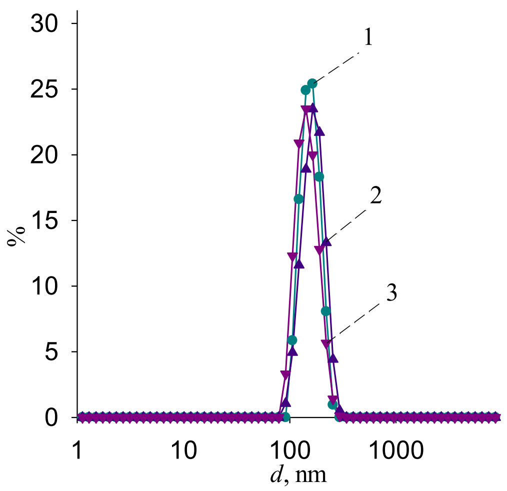

Fig. S17. Size distribution by intensity (1), volume (2), and particle number (3) of a 3:50 diluted initial system in DMSO.

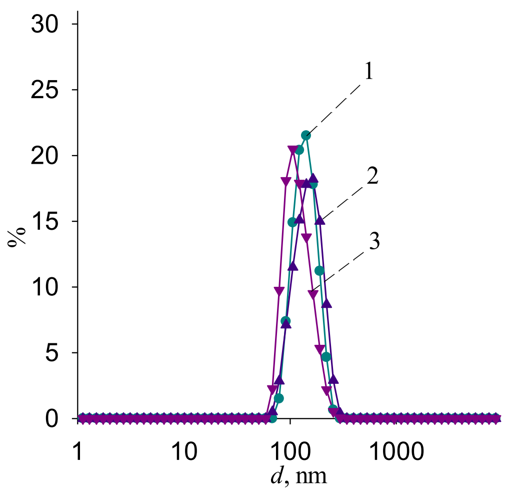

Fig. S18. Size distribution by intensity (1), volume (2), and particle number (3) of a 1:8 diluted initial system in DMSO.

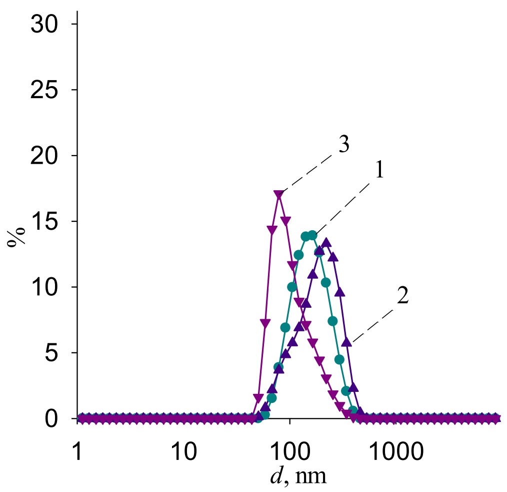

Fig. S19. Size distribution by intensity (1), volume (2), and particle number (3) of a 2:5 diluted initial system in DMSO.

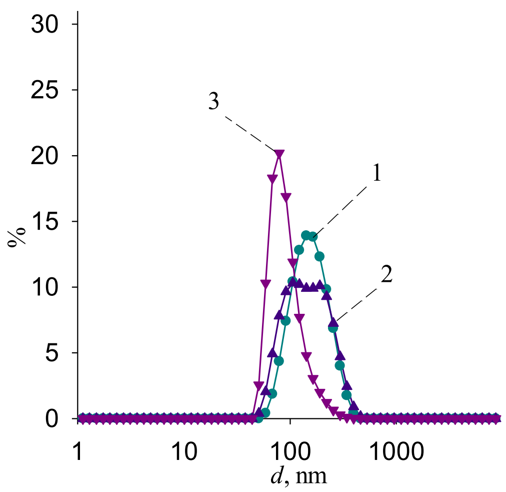

Fig. S20. Size distribution by intensity (1), volume (2), and particle number (3) of a 3:5 diluted initial system in DMSO.

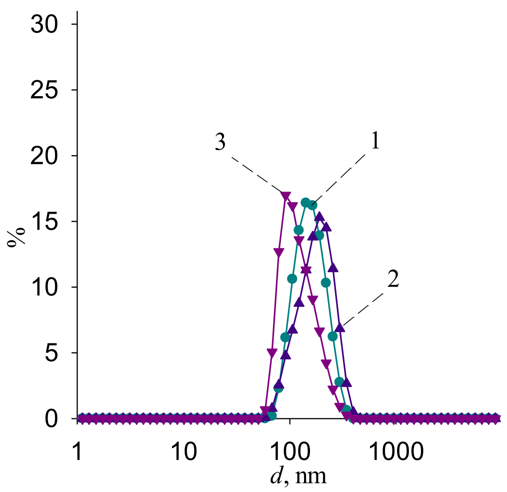

Fig. S21. Size distribution by intensity (1), volume (2), and particle number (3) of a 1:1 diluted initial system in DMSO.

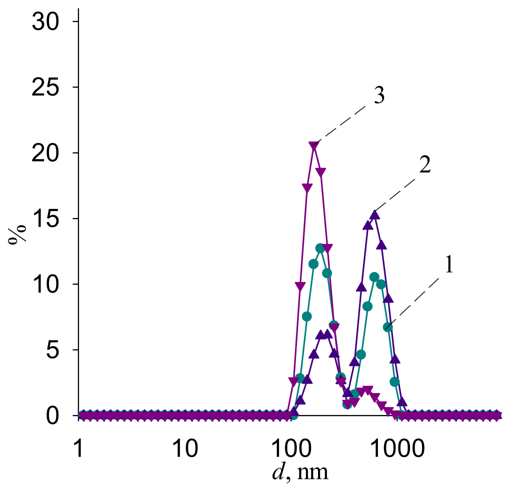

Fig. S22. Size distribution by intensity (1), volume (2), and particle number (3) of a 1:100 diluted initial system in DMSO with 90 v% H_2O.

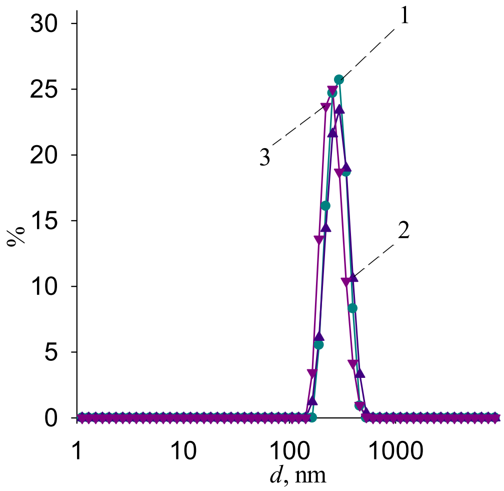

Fig. S23. Size distribution by intensity (1), volume (2), and particle number (3) of a 2:5 diluted initial system in DMSO with 60 v% H_2O.

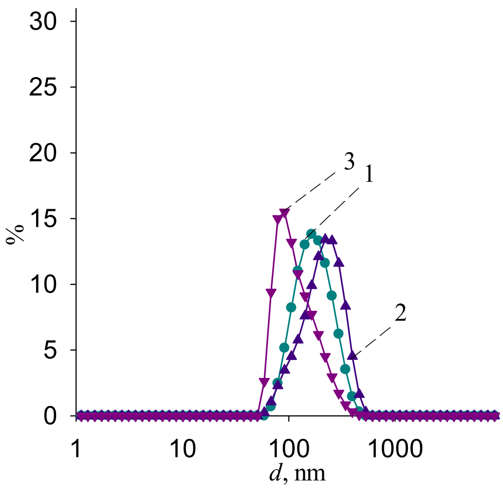

Fig. S24. Size distribution by intensity (1), volume (2), and particle number (3) of a 2:5 diluted initial system in DMSO with 5 v% H_2O.

Table S1. The zeta potential (ζ) by Huckel model and electrophoretic mobility of particles (u_e) in DMSO–H_2O systems at different TiO_2 dilution

System	ζ, mV	u_e, $\mu m \times cm \times V^{-1} \times c^{-1}$
1:100 diluted with 90 v% water	–(24 ± 2)	–(0.97 ± 0.07)
1:2.5 diluted with 60 v% water	10 ± 7	0.19 ± 0.09
1:2.5 diluted with 5 v% water	49 ± 1	0.61 ± 0.05

Table S2. Hydrodynamic size of particle and polydispersity index (PdI) in DMSO–H_2O systems at different TiO_2 dilution

System	PdI	Mean diameter, nm					
		Z_{ave}	by Intensity		by Volume		by Number
			I	II	I	II	
1:100 diluted with 90 v% water	0.636 ± 0.001	452.7 ± 0.1	196.3 ± 0.1	643 ± 20	212.8 ± 0.1	628 ± 10	208 ± 0.1
1:2.5 diluted with 60 v% water	0.013 ± 0.001	277.3 ± 0.1	286 ± 1	–	293.5 ± 0.1	–	259.4 ± 0.1
1:2.5 diluted with 5 v% water	0.131 ± 0.001	157.1 ± 0.1	182.3 ± 0.1	–	220.8 ± 0.1	–	125.6 ± 0.1

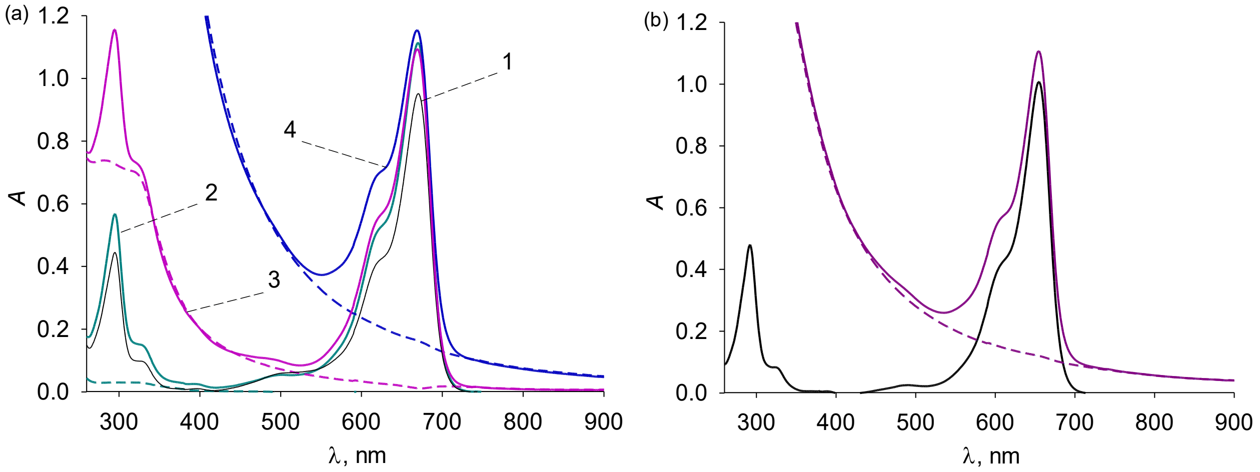

Fig. S25. Absorption spectra of MB in (a) DMSO: (1) without TiO_2, (2) TiO_2 (1:60), (3) TiO_2 (1:20), and (4) TiO_2 (1:3.5), and (b) ACN – TiO_2 (1:40). The blank solution was solvent. The dashed line represents systems without MB.

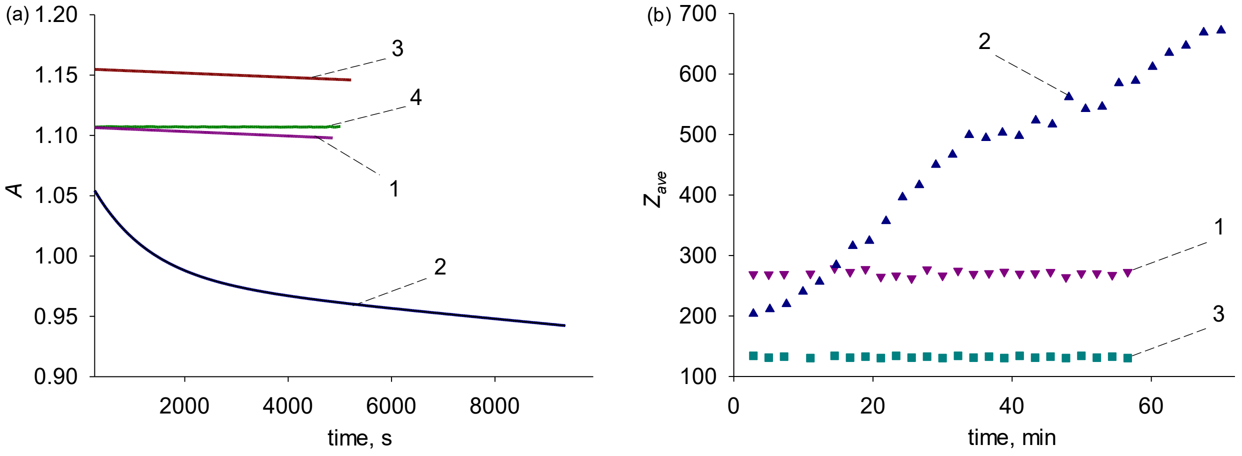

Fig. S26. Absorption and Z_{ave} vs time for MB in DMSO: (1) TiO_2 (1:60), (2) TiO_2 (1:20), (3) TiO_2 (1:3.5), and (4) ACN – TiO_2 (1:40) at 25 °C. The blank solution was solvent.

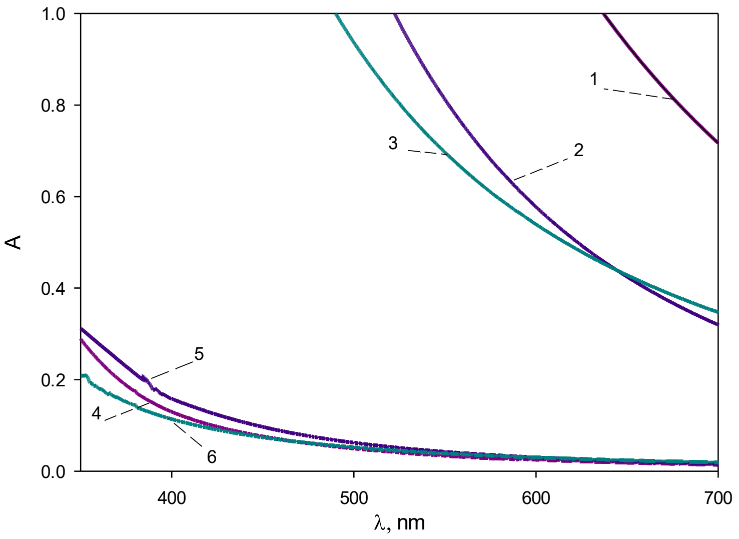

Fig. S27. Absorption spectra of created (1–3) and working (4–6) TiO_2 suspensions with LSZ (1 and 4), NaCh (2 and 5), and NaDCh (3 and 6).

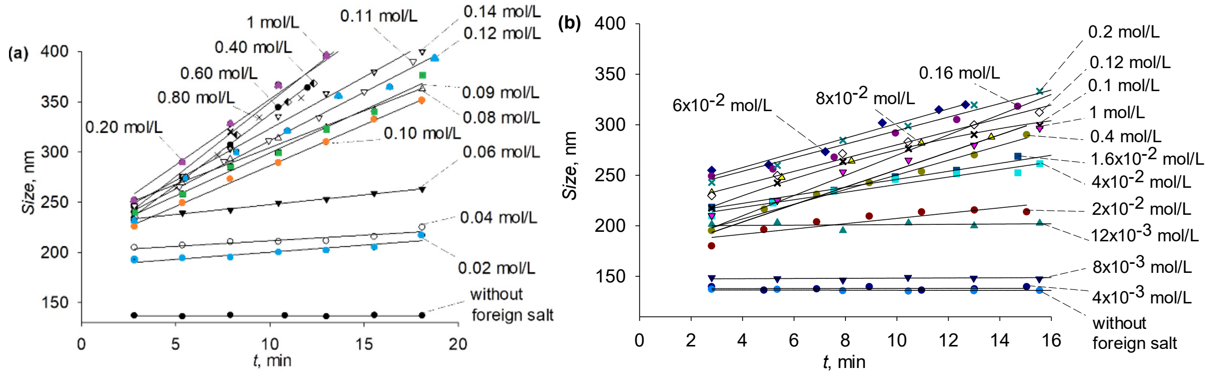

Fig. S28. Kinetics of particle aggregation at different concentrations of NaCl (a) and $CaCl_2$ (b) in aqueous dispersion of TiO_2 with LSZ.

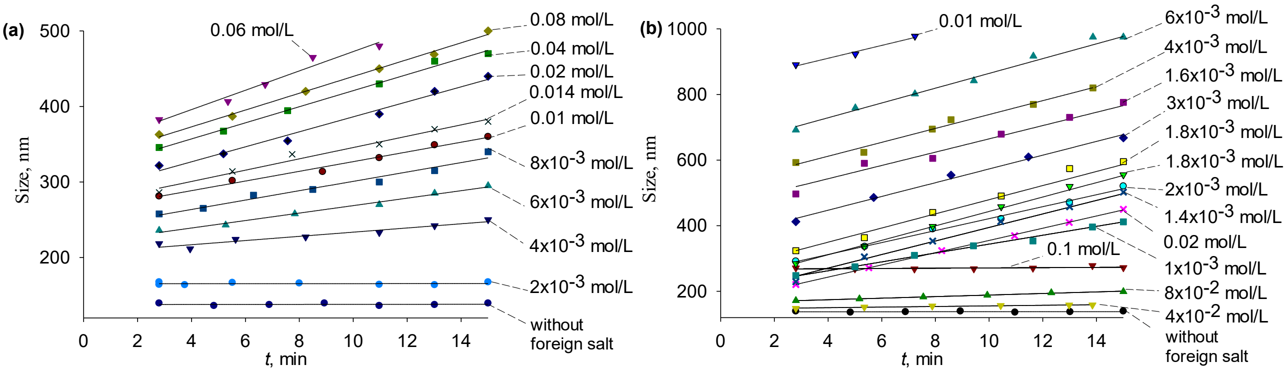

Fig. S29. Kinetics of particle aggregation at different concentrations of Na_2SO_4 (a) and NaCh (b) in aqueous dispersion of TiO_2 with LSZ.

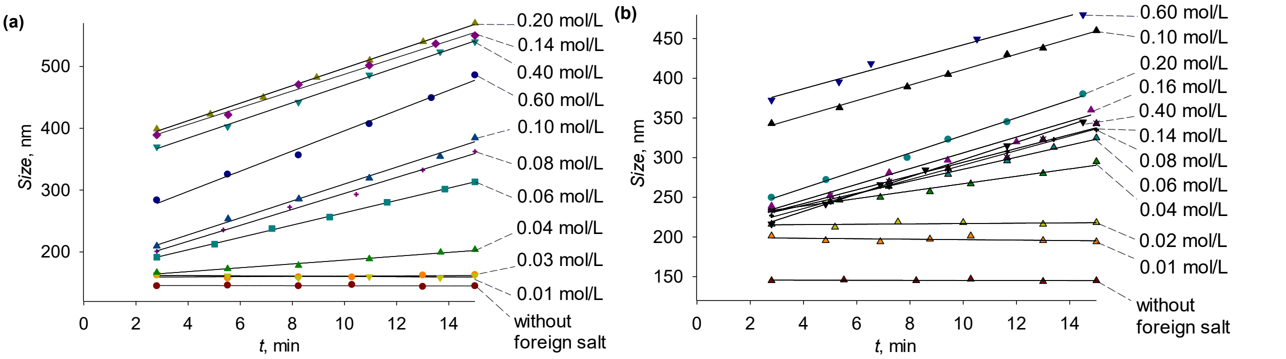

Fig. S30. Kinetics of particle aggregation at different concentrations of NaCl (a) and CsI (b) in aqueous dispersion of TiO_2 with NaCh.

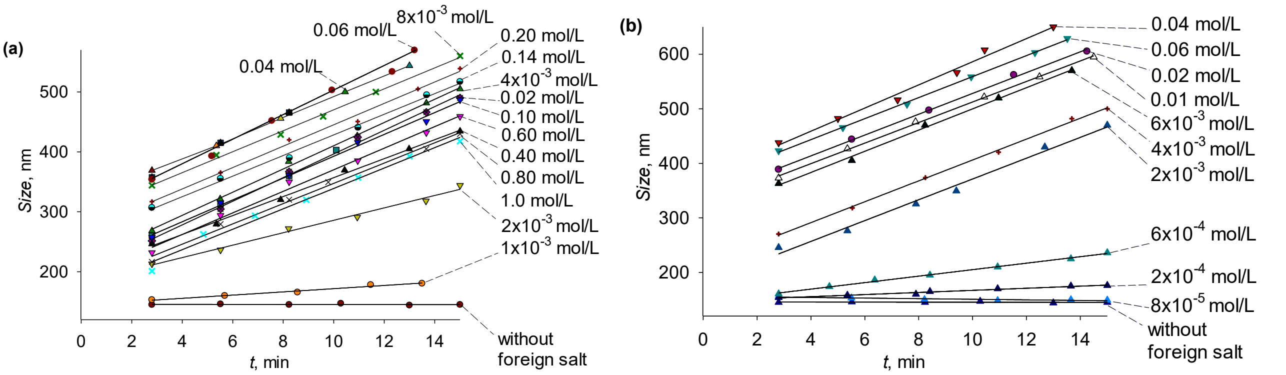

Fig. S31. Kinetics of particle aggregation at different concentrations of $CaCl_2$ (a) and $Sr(NO_3)_2$ (b) in aqueous dispersion of TiO_2 with NaCh.

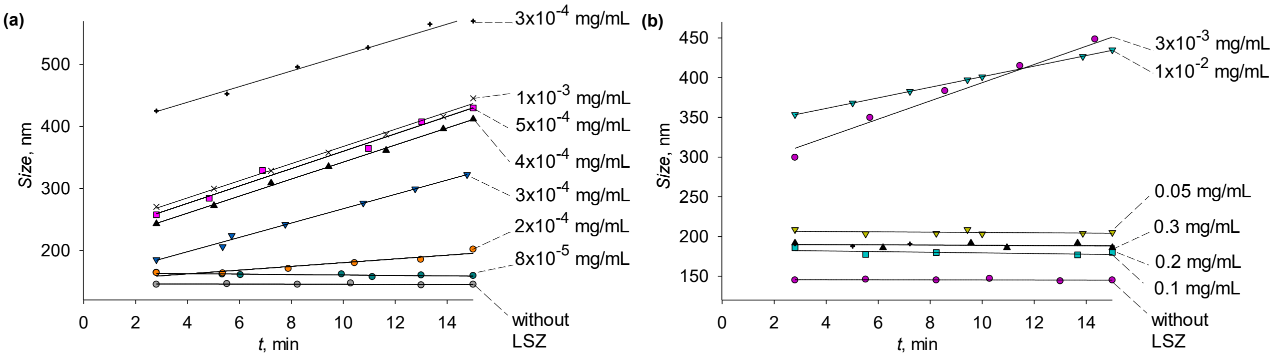

Fig. S32. Kinetics of particle aggregation at 8×10^{-5}–1×10^{-3} mg/mL (a) and 3×10^{-3}–1×10^{-2} mg/mL (b) LSZ in aqueous dispersion of TiO_2 with NaCh.

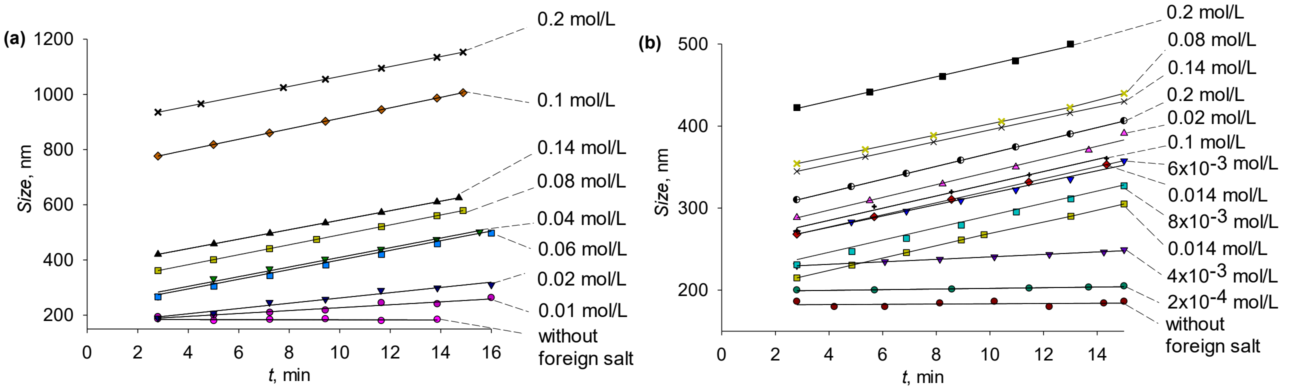

Fig. S33. Kinetics of particle aggregation at different concentrations of NaCl (a) and $CaCl_2$ (b) in aqueous dispersion of TiO_2 with NaDCh.
